# Identification of Chemosensory Genes Based on the Transcriptomic Analysis of Six Different Chemosensory Organs in *Spodoptera exigua*

**DOI:** 10.3389/fphys.2018.00432

**Published:** 2018-04-24

**Authors:** Ya-Nan Zhang, Jia-Li Qian, Ji-Wei Xu, Xiu-Yun Zhu, Meng-Ya Li, Xiao-Xue Xu, Chun-Xiang Liu, Tao Xue, Liang Sun

**Affiliations:** ^1^Department of Biological Sciences, College of Life Sciences, Huaibei Normal University, Huaibei, China; ^2^Key Laboratory of Tea Quality and Safety Control, Ministry of Agriculture, Tea Research Institute, Chinese Academy of Agricultural Sciences, Hangzhou, China

**Keywords:** *Spodoptera exigua*, olfactory organ, gustatory organ, transcriptome analysis, chemosensory gene

## Abstract

Insects have a complex chemosensory system that accurately perceives external chemicals and plays a pivotal role in many insect life activities. Thus, the study of the chemosensory mechanism has become an important research topic in entomology. *Spodoptera exigua* Hübner (Lepidoptera: Noctuidae) is a major agricultural polyphagous pest that causes significant agricultural economic losses worldwide. However, except for a few genes that have been discovered, its olfactory and gustatory mechanisms remain uncertain. In the present study, we acquired 144,479 unigenes of *S. exigua* by assembling 65.81 giga base reads from 6 chemosensory organs (female and male antennae, female and male proboscises, and female and male labial palps), and identified many differentially expressed genes in the gustatory and olfactory organs. Analysis of the transcriptome data obtained 159 putative chemosensory genes, including 24 odorant binding proteins (OBPs; 3 were new), 19 chemosensory proteins (4 were new), 64 odorant receptors (57 were new), 22 ionotropic receptors (16 were new), and 30 new gustatory receptors. Phylogenetic analyses of all genes and SexiGRs expression patterns using quantitative real-time polymerase chain reactions were investigated. Our results found that several of these genes had differential expression features in the olfactory organs compared to the gustatory organs that might play crucial roles in the chemosensory system of *S. exigua*, and could be utilized as targets for future functional studies to assist in the interpretation of the molecular mechanism of the system. They could also be used for developing novel behavioral disturbance agents to control the population of the moths in the future.

## Introduction

Over the evolutionary process, insects have developed a complex chemosensory system that can accurately perceive external chemicals. The system plays a pivotal role in many insect life activities, such as feeding, mating, host finding, searching for oviposition sites, avoiding predators, and migration (Field et al., 2000; Zhan et al., 2011; Suh et al., 2014; Sun et al., 2014; Zhang et al., 2015a). Numerous studies based on morphological and molecular biology have revealed that the antenna, proboscis, and labial palp are the main olfactory and gustatory organs in this system (Jacquin-Joly and Merlin, 2004; Briscoe et al., 2013; Sun et al., 2017).

The insect chemosensory system involves several different types of genes, including (1) soluble olfactory proteins in the lymph of chemosensilla, e.g., odorant binding proteins (OBPs) (Vogt, 2003; Xu et al., 2009; Zhou, 2010; Pelosi et al., 2018) and chemosensory proteins (CSPs) (Pelosi et al., 2005, 2006; Iovinella et al., 2013) that transfer chemicals via the chemosensilla lymph to corresponding chemosensory receptors, and (2) chemosensory membrane proteins, e.g., olfactory receptors (ORs) (Crasto, 2013; Leal, 2013; Zhang et al., 2015a, 2017), ionotropic receptors (IRs) (Vogt, 2003; Benton et al., 2009; Rytz et al., 2013), and gustatory receptors (GRs) (Clyne et al., 2000; Zhang et al., 2011; Briscoe et al., 2013; Ni et al., 2013) that are located on the dendrites of neurons in the chemosensilla and transform chemical signals into electrical signals to stimulate the corresponding behavioral responses of insects (Leal, 2013).

The acquisition, bioinformatics analysis, and expression pattern of putative chemosensory genes are the crucial steps to explore the exact roles of several key genes in the insect chemosensory process. The development of modern molecular biology techniques and experimental equipment, such as high-throughput sequencing, has created more efficient, inexpensive, and higher accuracy technologies than what has been traditionally utilized (McKenna et al., 1994; Picimbon and Gadenne, 2002; Xiu et al., 2008; Liu et al., 2012). These have been successfully applied in the identification of insect chemosensory genes, including many moth species, such as *Spodoptera littoralis* (Legeai et al., 2011), *Sesamia inferens* (Zhang et al., 2013), *Helicoverpa armigera* (Liu et al., 2014b), *Plutella xylostella* (Yang et al., 2017), and *Ectropis grisescens* (Li et al., 2017).

The beet armyworm, *Spodoptera exigua* Hübner (Lepidoptera: Noctuidae), is a major agricultural polyphagous pest that causes significant economic losses to many crops worldwide (Xiu and Dong, 2007; Acín et al., 2010; Lai and Su, 2011). To date, only partial chemosensory genes of *S. exigua* have been identified, including several OBPs (Xiu and Dong, 2007; Zhu et al., 2013; Liu et al., 2015b), CSPs (Liu et al., 2015b) and a few chemosensory receptor genes (Liu et al., 2013, 2014a, 2015b). This is much lower than other moth species from which chemosensory genes have been obtained from transcriptomic data of chemosensory organs. These limited gene resources impede our interpretation of the chemosensory molecular mechanism of *S. exigua*. To obtain greater olfactory and gustatory gene resources, we utilized the six major olfactory and gustatory organs (female antennae: FA, male antennae: MA, female proboscises: FPr, male proboscises: MPr, female labial palps: FLP, and male labial palps: MLP) of *S. exigua* adults in the present study. We first built a genetic database of genes that were expressed in the six chemosensory organs of *S. exigua* using an Illumina HiSeq™ 4000 sequencing platform and completely identified 159 genes (110 genes were newly obtained) as being potentially involved in the chemosensory system. To postulate the functions of these identified genes, we performed phylogenetic analyses of all genes and investigated SexiGRs expression patterns using quantitative real-time polymerase chain reaction (qPCR). Our results showed that several of the genes had differential expression in olfactory organs compared to gustatory organs that might play different and crucial roles in the chemosensory system of *S. exigua*, and could be utilized as targets for future functional studies (using the heterologous expression system of *Xenopus oocytes* or *Escherichia coli in vitro* and with genetic modification by the CRISPR/Cas9 editing system *in vivo*) to assist in the interpretation of the molecular mechanism of the system.

## Materials and methods

### Insects rearing and tissue collection

*S. exigua* larvae were purchased from Keyun Biology Company in Henan province, China. As we previous studies (Zhang et al., 2017a), we used same rearing conditions and methods to rear the insect. For transcriptome sequencing, 200 female antennae (FA), 200 male antennae (MA), 300 female proboscises (FPr), 300 male proboscises (MPr), 300 female labial palps (FLP), 300 male labial palps (MLP), 30 female abdomen (FAb), and 30 male abdomen (MAb) were collected from 3-day-old unmated adults. For the tissue distribution analysis, 100 FA, 100 MA, 200 FLP, 200 MLP, 200 FP, and 200 MP for each replicate experiment were collected under the same conditions. All these organs were immediately frozen in liquid nitrogen and stored at −80°C until use.

### cDNA library preparation, clustering, and sequencing

Sample total RNA was extracted using TRIzol reagent (Invitrogen, Carlsbad, CA, USA). cDNA library preparation and Illumina sequencing were carried out by Novogene Bioinformatics Technology Co., Ltd. (Beijing, China). The 1.5 μg total RNA per sample was used as input material for the RNA sample preparations, and sequencing libraries were generated using NEBNext® Ultra™ RNA Library Prep Kit for Illumina® (NEB, USA) following manufacturer's recommendations and index codes were added to attribute sequences to each sample. Briefly, mRNA was purified from total RNA using poly-T oligo-attached magnetic beads. Fragmentation was carried out using divalent cations under elevated temperature in NEBNext First Strand Synthesis Reaction Buffer (5X). First strand cDNA was synthesized using random hexamer primer and M-MuLV Reverse Transcriptase (RNase H-) (NEB, USA). Second strand cDNA synthesis was subsequently performed using DNA Polymerase I (NEB, USA) and RNase H (NEB, USA). Remaining overhangs were converted into blunt ends via exonuclease/polymerase activities. After adenylation of 3′ ends of DNA fragments, NEBNext Adaptor with hairpin loop structure were ligated to prepare for hybridization. In order to select cDNA fragments of preferentially 150~200 bp in length, the library fragments were purified with AMPure XP system (Beckman Coulter, Beverly, USA). Then 3 μL USER Enzyme (NEB, USA) was used with size-selected, adaptor-ligated cDNA at 37°C for 15 min followed by 5 min at 95°C before PCR. Then PCR was performed with Phusion High-Fidelity DNA polymerase, Universal PCR primers, and Index (X) Primer. At last, PCR products were purified (AMPure XP system) and library quality was assessed on the Agilent Bioanalyzer 2100 system.

The clustering of the index-coded samples was performed on a cBot Cluster Generation System using TruSeq PE Cluster Kit v3-cBot-HS (Illumina, San Diego, CA, USA) according to the manufacturer's instructions. After cluster generation, the library preparations were sequenced on an Illumina Hiseq™ 4000 platform and paired-end reads were generated.

### Transcriptome assembly and gene functional annotation

Transcriptome assembly was accomplished based on the reads using Trinity (r20140413p1) (Li et al., 2010; Grabherr et al., 2011) with min_kmer_cov set to 2 by default and all other parameters set default. The assembly sequences of Trinity were deemed to be unigenes. Unigene function was annotated based on the following databases: Nr (NCBI non-redundant protein sequences) (https://www.ncbi.nlm.nih.gov/genbank/ and https://www.ncbi.nlm.nih.gov/protein/), Pfam (Protein family) (https://pfam.sanger.ac.uk/), KOG/COG (Clusters of Orthologous Groups of proteins) (https://www.ncbi.nlm.nih.gov/COG/), Swiss-Prot (A manually annotated and reviewed protein sequence database) (http://www.ebi.ac.uk/uniprot/), KO (KEGG Ortholog database) (http://www.genome.jp/kegg/) and GO (Gene Ontology) (http://www.geneontology.org/).

### Differential expression analysis

Firstly, the read counts were adjusted by edgeR 3.0.8 program package through one scaling normalized factor for each sequenced library. Then, the differential expression analysis of two samples was performed using the DEGseq 1.12.0 R package (Wang et al., 2010). *P*-value was adjusted using *q*-value (Storey, 2003). *q* < 0.005 & |log2(foldchange)|>1 was set as the threshold for significantly differential expression.

### RNA isolation and cDNA synthesis

Total RNA was extracted using the MiniBEST Universal RNA Extraction Kit (TaKaRa, Dalian, China), following the manufacturer's instructions, in which we used DNase I to digest sample DNase to avoid genomic DNA contamination. The RNA quality was assessed spectrophotometrically (Biofuture MD2000D, UK). Single-stranded cDNA templates were synthesized from 1 μg total RNA obtained from various tissue samples using the PrimeScript™ RT Master Mix (TaKaRa, Dalian, China) according to the manufacturers' instructions.

### Sequence and phylogenetic analysis

The ORFs of the chemosensory genes were predicted by using ORF Finder (http://www.ncbi.nlm.nih.gov/gorf/gorf.html), and the similarity searches of genes were performed by using the NCBI-BLAST Server (http://blast.ncbi.nlm.nih.gov/). Putative N-terminal signal peptides (SP) of SexiOBPs and SexiCSPs were predicted by SignalP 4.1 (http://www.cbs.dtu.dk/services/SignalP/) (Petersen et al., 2011). Transmembrane domains (TMD) of SexiORs, SexiGRs, and SexiIRs were predicted by TMHMM Server Version 2.0 (Krogh et al., 2001) (http://www.cbs.dtu.dk/services/TMHMM).

Phylogenetic trees were constructed for the analysis of five family chemosensory genes of *S. exigua*, based on gene sequences of *S. exigua* and those of other insects. The OBP data set contained 24 sequences from *S. exigua* (Table S1), and 90 from other species, including *B. mori* (Gong et al., 2009), *M. sexta* (Grosse-Wilde et al., 2011), and *A. lepigone* (Zhang et al., 2017b). The CSP data set contained 19 sequences from *S. exigua* (Table S1), and 55 from other species, including *B. mori* (Gong et al., 2007), *M. sexta* (Grosse-Wilde et al., 2011), and *A. lepigone* (Zhang et al., 2017b). The OR data set contained 64 sequences from *S. exigua* (Table S1), and 91 from other species (Tanaka et al., 2009; Zhan et al., 2011; Zhang et al., 2015b). The IR data set contained 22 sequences from *S. exigua* (Table S1), and 131 from other species (Croset et al., 2010; Olivier et al., 2011; Rimal and Lee, 2018). The GR data set contained 30 sequences from *S. exigua* (Table S1), and 126 from other species (Zhan et al., 2011; Liu et al., 2014b; Guo et al., 2017). Then, we used ClustalX 1.83 (Larkin et al., 2007) to align amino acid sequences from the same family gene, and used PhyML 3.1 (Guindon et al., 2010) based on the LG substitution model (Le and Gascuel, 2008) with Nearest Neighbor Interchange (NNI) to construct the phylogenetic trees, and the branch support of tree estimated by a Bayesian-like transformation of the aLRT (aBayes) method (Anisimova et al., 2011). Lastly, we created and edited the different trees by using the FigTree 1.4.2 software (http://tree.bio.ed.ac.uk/software/figtree/).

### Quantitative real-time PCR (qPCR) analysis

According to the minimum information for publication of qPCR experiments (Bustin et al., 2009) and our previous studies (Zhang et al., 2017a), we performed the qPCR assay of tissue distribution of SexiGRs in ABI 7300 (Applied Biosystems, Foster City, CA, USA) by using 2×SYBR Green PCR Master Mix (YIFEIXUE BIO TECH, Nanjing, China) as the manufacturer's instructions. Briefly, the reaction programs were 10 min at 95°C, 40 cycles of 95°C for 15 s and 60°C for 1 min. The qPCR primers (Table S2) were designed using Beacon Designer 7.9 (PREMIER Biosoft International, CA, USA). Then, the relative expression levels of SexiGRs mRNA were calculated based on the Ct-values of target gene and two reference genes SexiGAPDH (glyceraldehyde-3-phosphate dehydrogenase) and SexiEF (elongation factor-1 alpha) by using the Q-Gene method in Microsoft Excel-based software of Visual Basic (Muller et al., 2002; Simon, 2003), the qPCR data are listed in Table S3. To ensure the reliability of the results, we carried out three biological replications for each sample and three technical replications for each biological replication.

### Statistical analysis

Data (mean ± SE) from various samples were subjected to one-way nested analysis of variance (ANOVA), followed by the least significant difference test (LSD) for comparison of means using SPSS Statistics 22.0 (SPSS Inc., Chicago, IL, USA).

## Results and discussion

### Overview of transcriptomes from the six organs

**Figure 1:**
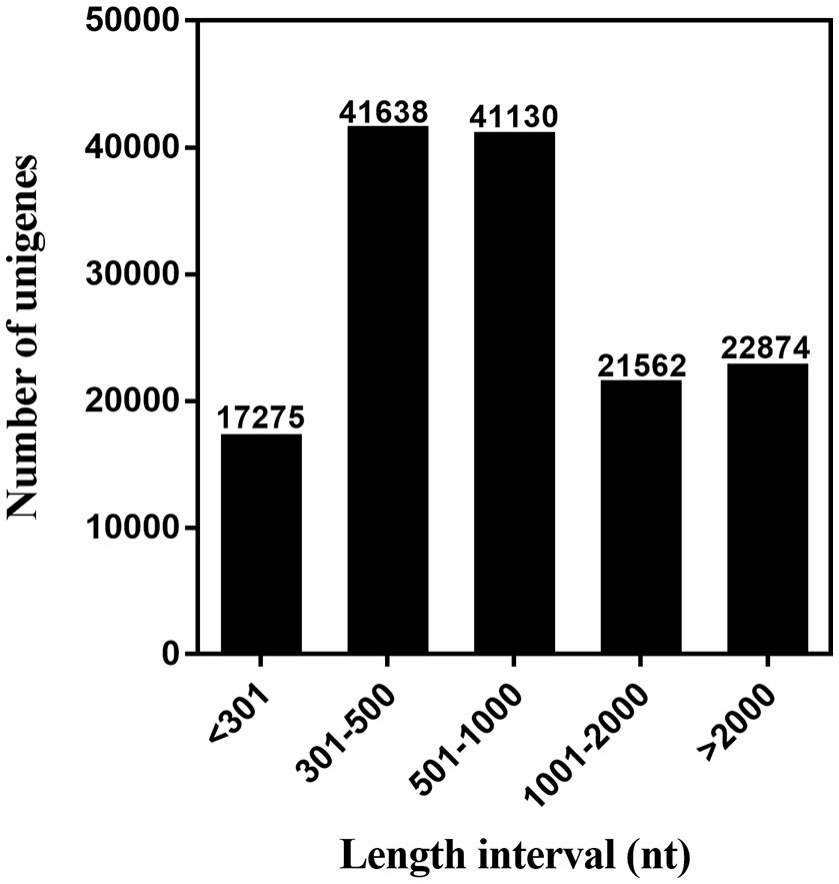
Distribution of unigene size in the *S. exigua* transcriptome assembly.

We used next-generation sequencing to sequence the six cDNA libraries constructed from the chemosensory organs (FA, MA, FPr, MPr, FLP, and MLP) of *S. exigua* adults based on the Illumina HiSeq™ 4000 platform and acquired 65.81 (from 10.60 to 11.90) giga base reads. After clustering and redundancy filtering, we finally obtained 144,479 unigenes and 266,645 transcripts with a N50 length of 2,177 base pair (bp) and 1,552 bp, respectively (Table 1). Statistics showed that 59.22% of the 144,479 unigenes were greater than 500 bp in length (Figure 1). The number of reads, unigenes, and transcripts were higher than most other insects based on transcriptome studies.

**Table 1:** Summary of *S. exigua* transcriptome assembly.

**Sample name**	**FA**	**MA**	**FPr**	**MPr**	**FLP**	**MLP**
Total size (Gb)	10.61	11.27	11.90	10.69	10.74	10.60
GC percentage (%)	43.58	42.93	45.13	45.00	46.80	46.35
Q20 percentage (%)	95.95	96.01	96.53	96.90	94.92	96.49
Number of transcripts			266,645		
Total unigene			144,479			
Total transcript nucleotides			202,244,136			
Total unigene nucleotides			168,211,374			
N50 of transcripts (nt)			1,552			
N50 of unigenes (nt)			2,177			
Max length of unigenes (nt)			30,184			
Min length of unigenes (nt)			201			
Median length of unigenes (nt)			584			
Unigenes with homolog in NR			60,373			

**Figure 2:**
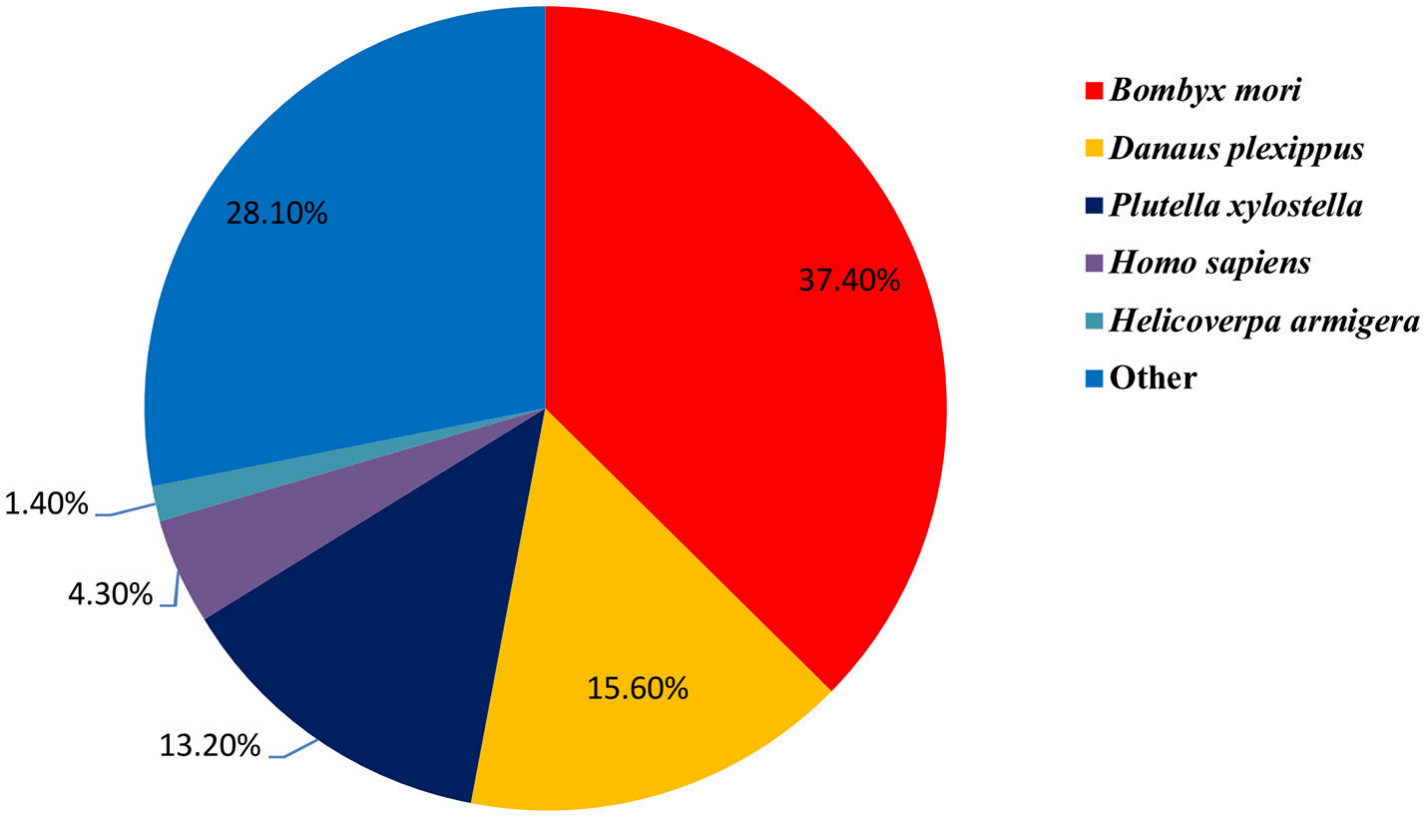
Percentage of homologous hits of the *S. exigua* unigenes to other insect species. The *S. exigua* transcripts were searched by Blastx against the Nr protein database with a cutoff *E*-value 10^−5^. Species that have more than 1% matching hits to the *S. exigua* transcripts are shown.

In total, 60,373 unigenes were matched to entries in the National Center for Biotechnology Information (NCBI) non-redundant (NR) protein database (http://www.ncbi.nlm.nih.gov/protein) by a BLASTX homology search with a cut-off *e*-value of 10^−5^. The highest match percentage (37.40%) was identified with sequences of *Bombyx mori* followed by sequences of *Danaus plexippus* (15.60%), *P. xylostella* (13.20%), *Homo sapiens* (4.30%), and *H. armigera* (1.40%; Figure 2).

**Figure 3:**
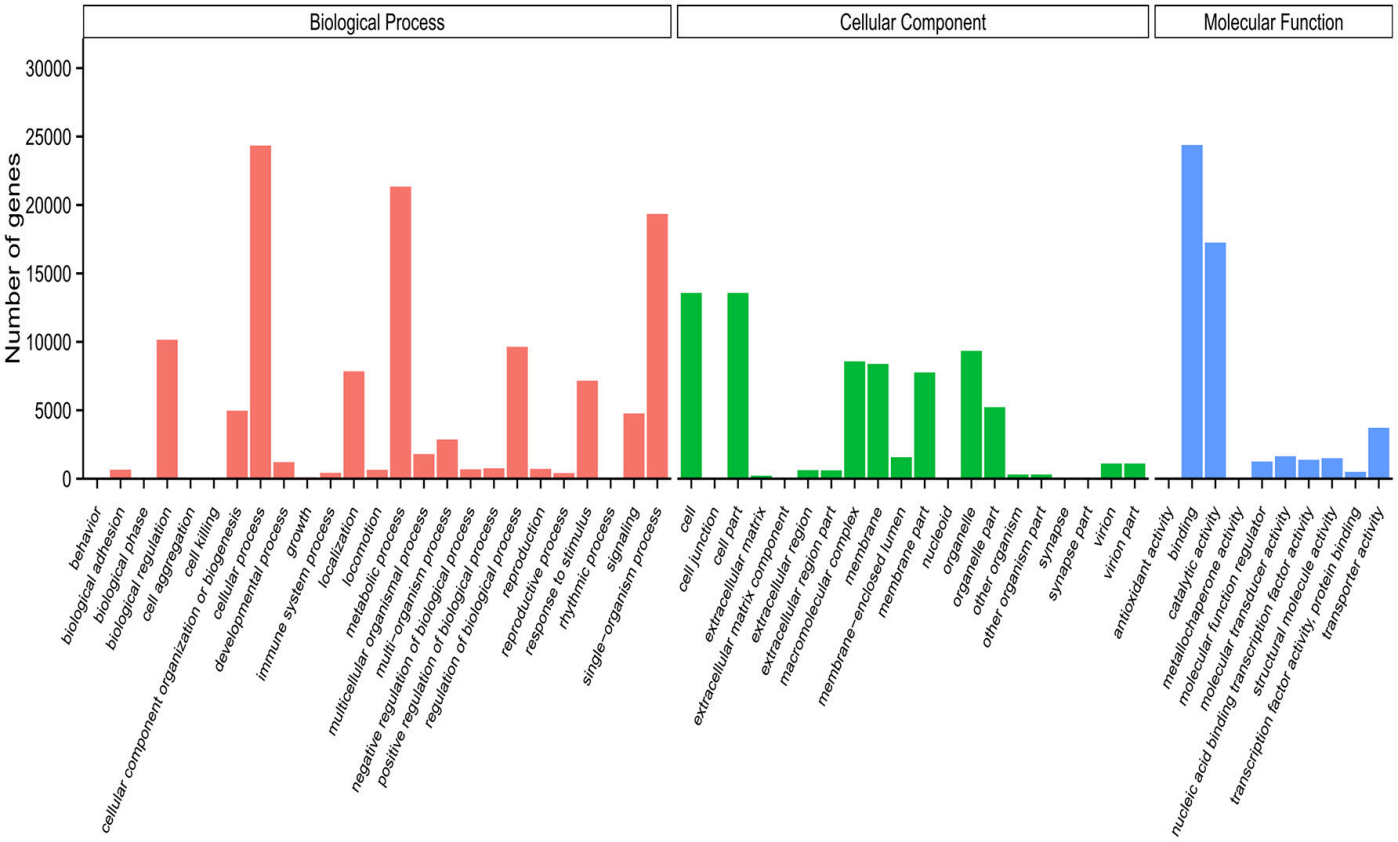
Gene ontology (GO) classification of the *S. exigua* unigenes with Blast2GO program.

Based on methodology described in our previous studies (Zhang et al., 2013; Li et al., 2015), we applied Blast2GO to classify the functional groups of all unigenes. The results showed that only 29.29% (42,331) of the 144,479 unigenes could be annotated based on the sequence homology, with this proportion similar to that found in other insects (Gu et al., 2013; Zhang et al., 2013; He et al., 2017). One possible reason for this might be that a great amount of *S. exigua* unigenes belong to non-coding or homologous genes without a gene ontology (GO) term. In addition, the GO annotation of *S. exigua* unigenes displayed similar classification to the unigenes of chemosensory organs from other moth species (Grosse-Wilde et al., 2011; Zhang et al., 2013; Cao et al., 2014; Xia et al., 2015). For example, unigenes of *S. exigua* during biological processes were predicted to be mostly enriched in three sub-categories: cellular, metabolic, and single-organism processes. There was also expected to be similarity in the cellular components (e.g., cell, cell part, and organelle) and molecular function categories (binding, catalytic, and transporter activity; Figure 3), indicating that some unigenes in these sub-categories might play important roles in the chemosensory behavior of moths.

### Differentially expressed genes (DEGs)

**Figure 4:**
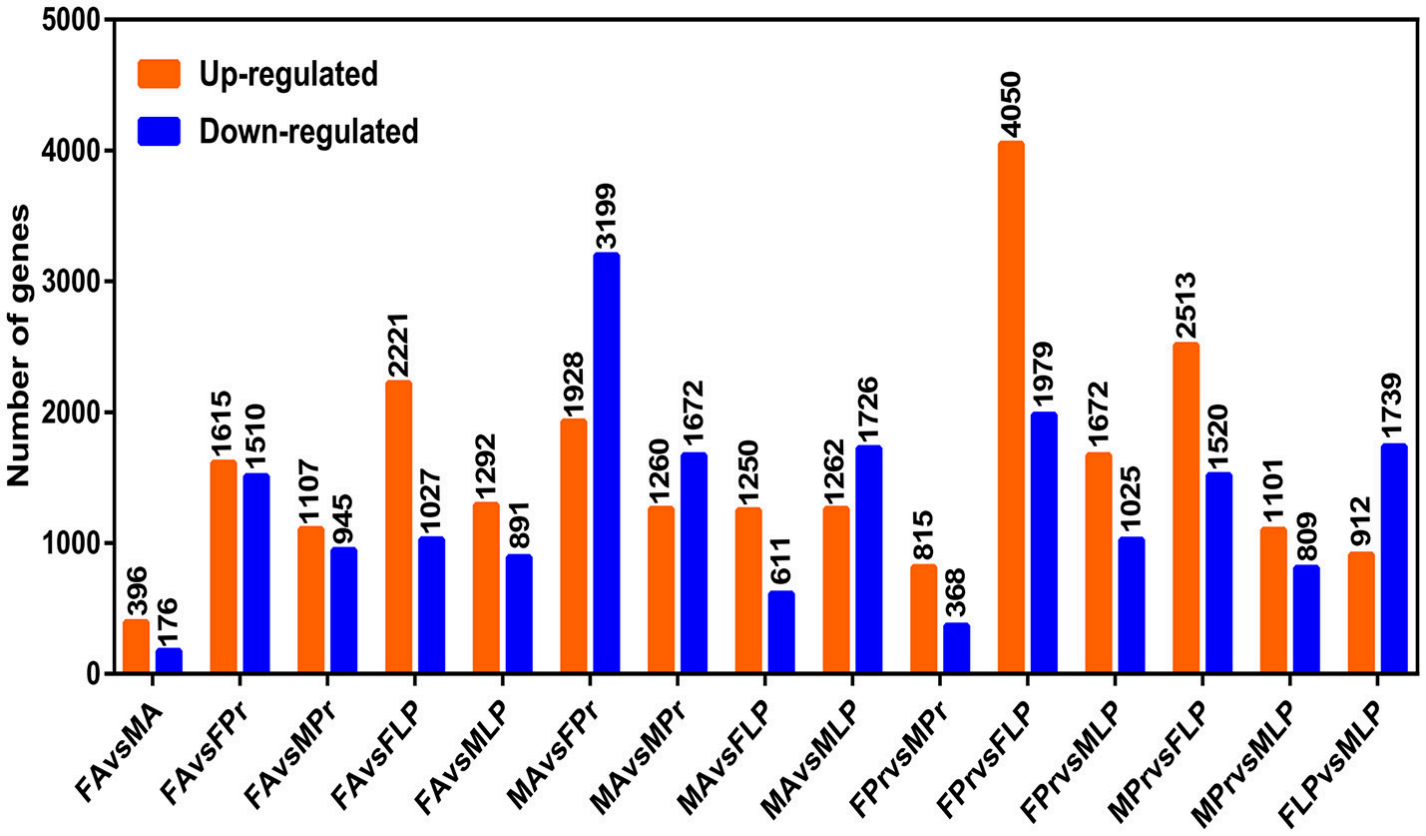
Differentially expressed genes (DEGs) among the different organs of *S. exigua*. FA, female antennae; MA, male antennae; FLP, female labial palps; MLP, male labial palps; FPr, female proboscises; MPr, male proboscises.

To investigate the DEGs among different organs, we compared each organ pair-wise within each sex against all other organs (Figure 4). Gene expression dynamics can be reflected by up- or down-regulation among the six different organs by pairwise comparisons. The results showed that there were a number of DEGs between different organs and different sexes, and the number of DEGs was highest in FPr vs. FLP (6,029 genes in total: 4,050 up-regulated genes and 1,979 down-regulated genes), followed by MA vs. FPr (5,127 genes in total: 1,928 up-regulated genes and 3,199 down-regulated genes), and MPr vs. FLP (4,033 genes in total: 2,513 up-regulated genes and 1,520 down-regulated genes). This indicates that these DEGs, especially in the gustatory vs. olfactory organs, provide substantial genetic sources that are important for studying the differential mechanism of gustatory vs. olfactory organs in *S. exigua*. Additionally, they provide some important target genes to analyse the functions of expressed sex-specific genes to reveal sex differences in chemosensory mechanisms in the future.

### Identification of putative chemosensory genes

Based on sequence similarity analyses and characteristics of insect chemosensory genes from previous studies (Xu et al., 2009; Croset et al., 2010; Zhou, 2010; Zhang et al., 2011; Ray et al., 2014), such as the conserved C-pattern of OBPs and CSPs, and the conserved transmembrane structure and motifs of chemosensory receptors (ORs, IRs, and GRs), we totally identified 159 putative genes from the transcriptomic data of *S. exigua* chemosensory organs that belonged to five insect chemosensory gene families. These included 24 OBPs, 19 CSPs, 64 ORs, 22 IRs, and 30 GRs (Tables 2, 3). The number of putative chemosensory genes of *S. exigua* identified in the present study was higher than that in other moth species where the same family genes had been identified by analysis of the transcriptome of specific organs. This included *H. armigera* (143 genes: 34 OBPs, 18 CSPs, 60 ORs, 21 IRs, and 10 GRs) (Liu et al., 2014b), *H. assulta* (147 genes: 29 OBPs, 17 CSPs, 64 ORs, 19 IRs, and 18 GRs) (Xu et al., 2015), and *P. xyllostella* (116 genes: 24 OBPs, 15 CSPs, 54 ORs, 16 IRs, and 7 GRs) (Yang et al., 2017). We found that the amount of transcriptomic data of these three different moth species was less than that of *S. exigua* in the present study, which suggests that the large amount of transcriptomic data could help us obtain more insect chemosensory genes.

**Table 2:** The Blastx match of *S. exigua* putative OBP and CSP genes.

**Gene**	**ORF**	**Signal**	**Complete**	**Best Blastx Match**				
**Name**	**(aa)**	**Peptide**	**ORF**	**Name**	**Acc. No**.	**Species**	***E*-value**	**Identity (%)**
**ODORANT BINDING PROTEIN (OBP)**								
PBP1	164	1–23	Y	Pheromone binding protein 1	AAS46620.1	*Spodoptera exigua*	1.00E-66	100
PBP2	170	1–27	Y	Pheromone binding protein 2	AAS55551.2	*Spodoptera exigua*	5.00E-82	100
PBP3	164	1–22	Y	Pheromone binding protein 3	ACY78413.1	*Spodoptera exigua*	2.00E-110	100
GOBP1	164	1–19	Y	General binding protein 1	ACY78412.1	*Spodoptera exigua*	2.00E-84	100
GOBP2	162	1–17	Y	Odorant binding protein 2	AGH70098.1	*Spodoptera exigua*	5.00E-28	100
OBP1	147	1–21	Y	Odorant binding protein 1	ADY17883.1	*Spodoptera exigua*	1.00E-29	100
OBP2	133	1–17	Y	Odorant binding protein 2	ADY17884.1	*Spodoptera exigua*	4.00E-69	100
OBP4	145	1–17	Y	Odorant binding protein 4	ADY17886.1	*Spodoptera exigua*	1.00E-96	100
OBP5	121	N	Y	Odorant binding protein 5	AFM77983.1	*Spodoptera exigua*	4.00E-75	100
OBP7	157	1–20	Y	Odorant binding protein 7	ADY17882.1	*Spodoptera exigua*	2.00E-105	100
OBP7	142	1–21	Y	Odorant binding protein 7	AGH70103.1	*Spodoptera exigua*	2.00E-92	100
OBP8	149	1–26	Y	Odorant binding protein 8	AGH70104.1	*Spodoptera exigua*	3.00E-25	100
OBP9	133	1–16	Y	Odorant binding protein 9	AGH70105.1	*Spodoptera exigua*	8.00E-48	100
OBP11	173	N	Y	Odorant binding protein 11	AGH70107.1	*Spodoptera exigua*	2.00E-88	100
OBP12	145	1–24	Y	SexiOBP12	AGP03458.1	*Spodoptera exigua*	8.00E-71	100
OBP17	148	1–17	Y	Odorant binding protein 17	AKT26495.1	*Spodoptera exigua*	1.00E-79	100
OBP18	186	1–17	Y	Odorant binding protein 18	AKT26496.1	*Spodoptera exigua*	2.00E-52	100
OBP24	184	1–20	Y	Odorant binding protein 24	AKT26501.1	*Spodoptera exigua*	6.00E-45	100
OBP25	239	1–19	Y	Odorant binding protein 25	AKT26502.1	*Spodoptera exigua*	2.00E-166	100
OBP27	118	N	Y	Odorant binding protein 27	AKT26504.1	*Spodoptera exigua*	9.00E-57	100
ABP	147	1–21	Y	Antennal binding protein	ADY17881.1	*Spodoptera exigua*	2.00E-59	100
OBP-N1	137	1–19	Y	General odorant-binding protein 69a-like	XP_022827633.1	*Spodoptera litura*	1.00E-68	97
OBP-N2	110	N	N	Odorant binding protein OBP6	ALJ30193.1	*Spodoptera litura*	7.00E-17	37
OBP-N3	127	1–21	Y	Odorant binding protein 6	AKI87967.1	*Spodoptera litura*	1.00E-71	99
**CHEMOSENSORY PROTEIN (CSP)**								
CSP1	128	1–18	Y	Chemosensory protein 1	ABM67688.1	*Spodoptera exigua*	8.00E-82	100
CSP2	128	1–18	Y	Chemosensory protein CSP2	ABM67689.1	*Spodoptera exigua*	9.00E-72	100
CSP3	126	1–16	Y	Chemosensory protein CSP3	ABM67690.1	*Spodoptera exigua*	7.00E-77	100
CSP4	123	1–18	Y	Chemosensory protein CSP4	AKT26481.1	*Spodoptera exigua*	2.00E-80	100
CSP5	131	1–25	Y	Chemosensory protein 5	AKT26482.1	*Spodoptera exigua*	2.00E-69	98
CSP6	127	1–17	Y	Chemosensory protein 6	AKT26483.1	*Spodoptera exigua*	3.00E-69	100
CSP7	128	1–16	Y	Chemosensory protein 7	AKT26484.1	*Spodoptera exigua*	4.00E-20	100
CSP8	107	1–17	Y	Chemosensory protein 8	AKT26485.1	*Spodoptera exigua*	9.00E-52	100
CSP10	122	1–19	Y	Chemosensory protein 10	AKT26486.1	*Spodoptera exigua*	7.00E-72	100
CSP11	122	1–16	Y	Chemosensory protein 11	AKT26487.1	*Spodoptera exigua*	2.00E-30	100
CSP12	125	1–15	Y	Chemosensory protein 12	AKT26488.1	*Spodoptera exigua*	1.00E-62	100
CSP13	123	1–16	Y	Chemosensory protein CSP13	AKT26489.1	*Spodoptera exigua*	2.00E-74	100
CSP14	287	1–16	Y	Chemosensory protein 14	AKT26490.1	*Spodoptera exigua*	1.00E-40	100
CSP19	122	1–17	Y	Chemosensory protein 19	AKT26493.1	*Spodoptera exigua*	7.00E-71	100
CSP20	107	1–18	Y	Chemosensory protein 20	AKT26494.1	*Spodoptera exigua*	3.00E-54	100
CSP-N1	148	1–21	Y	Chemosensory protein 4	AND82446.1	*Athetis dissimilis*	5.70E-71	77
CSP-N2	123	1–18	Y	Putative chemosensory protein CSP3	ALJ30214.1	*Spodoptera litura*	7.00E-79	99
CSP-N3	98	N	N	Chemosensory protein CSP	AAY26143.1	*Spodoptera litura*	1.00E-65	100
CSP-N4	123	1–16	Y	Putative chemosensory protein CSP6	ALJ30217.1	*Spodoptera litura*	8.00E-75	99

**Table 3:** The Blastx Match of *S. exigua* putative OR, IR and GR genes.

**Gene**	**ORF**	**TMD**	**Complete**	**Best Blastx Match**				
**Name**	**(aa)**		**ORF**	**Name**	**Acc. No**.	**Species**	***E*-value**	**Identity (%)**
**ODORANT RECEPTOR (OR)**								
Orco	473	7	Y	Putative chemosensory receptor 2	AAW52583.1	*Spodoptera exigua*	0.00E+00	100
OR1	290	–	N	Putative odorant receptor OR61	AOE48066.1	*Athetis lepigone*	0.00E+00	79
OR2	415	6	Y	Putative odorant receptor OR25	AOE48030.1	*Athetis lepigone*	0.00E+00	70
OR3	413	7	Y	Odorant receptor	AEF32141.1	*Spodoptera exigua*	0.00E+00	99
OR4	130	–	N	Putative olfactory receptor 51	AGG08876.1	*Spodoptera litura*	7.00E−86	72
OR5	114	–	N	Odorant receptor	AIG51858.1	*Helicoverpa armigera*	6.00E−60	83
OR6	432	5	Y	Odorant receptor 6	AGH58119.1	*Spodoptera exigua*	0.00E+00	99
OR7	442	6	Y	Olfactory receptor 2	JAV45863.1	*Mythimna separata*	0.00E+00	86
OR8	60	–	N	Olfactory receptor 24	AQQ73504.1	*Heliconius melpomene rosina*	1.00E−09	58
OR9	312	–	N	Putative chemosensory receptor 9	CAD31950.1	*Heliothis virescens*	7.00E−122	64
OR10	402	6	Y	Odorant receptor	AIG51887.1	*Helicoverpa armigera*	0.00E+00	87
OR11	435	7	Y	Odorant receptor 11	AGH58120.1	*Spodoptera exigua*	0.00E+00	100
OR12	418	6	Y	Odorant receptor 50	KOB74670.1	*Operophtera brumata*	4.00E−144	51
OR13	445	5	Y	Odorant receptor 13	AGH58121.1	*Spodoptera exigua*	0.00E+00	99
OR14	393	6	Y	Odorant receptor	AIG51868.1	*Helicoverpa armigera*	0.00E+00	80
OR15	247	–	N	Putative odorant receptor OR44	AOE48049.1	*Athetis lepigone*	1.00E−156	90
OR16	432	4	Y	Odorant receptor 16	AGH58122.1	*Spodoptera exigua*	0.00E+00	99
OR17	207	–	N	Odorant receptor	AIG51882.1	*Helicoverpa armigera*	7.00E−142	76
OR18	94	–	N	Putative odorant receptor OR56	AOE48061.1	*Athetis lepigone*	4.00E−22	68
OR19	303	–	N	Odorant receptor 15	ALM26204.1	*Athetis dissimilis*	1.00E−163	70
OR20	399	5	Y	Putative odorant receptor OR27	AOE48032.1	*Athetis lepigone*	0.00E+00	81
OR21	418	5	Y	Odorant receptor 38	ALM26228.1	*Athetis dissimilis*	0.00E+00	87
OR22	320	–	N	Olfactory receptor 11	JAV45854.1	*Mythimna separata*	0.00E+00	86
OR23	414	6	Y	Odorant receptor 50	KOB74670.1	*Operophtera brumata*	0.00E+00	64
OR24	381	6	Y	Odorant receptor	AIG51892.1	*Helicoverpa armigera*	0.00E+00	81
OR25	235	–	N	Odorant receptor	AIG51900.1	*Helicoverpa armigera*	1.00E−126	85
OR26	289	–	N	Putative odorant receptor OR12	AOE48017.1	*Athetis lepigone*	2.00E−121	75
OR27	236	–	N	Olfactory receptor 17	AGK90007.1	*Helicoverpa armigera*	2.00E−111	74
OR28	134	–	N	Putative odorant receptor SinfOR18	AIF79425.1	*Sesamia inferens*	3.00E−71	85
OR29	373	5	Y	Odorant receptor	AIG51879.1	*Helicoverpa armigera*	0.00E+00	83
OR30	351	5	Y	Putative odorant receptor OR23	AOE48028.1	*Athetis lepigone*	0.00E+00	75
OR31	263	–	N	Putative olfactory receptor 12	AGG08878.1	*Spodoptera litura*	0.00E+00	97
OR32	156	–	N	Odorant receptor	AIG51886.1	*Helicoverpa armigera*	2.00E−80	77
OR33	240	–	N	Odorant receptor 37	ALM26227.1	*Athetis dissimilis*	2.00E−161	59
OR34	146	–	N	Olfactory receptor 41	JAV45824.1	*Mythimna separata*	8.00E−81	83
OR35	366	6	Y	Putative olfactory receptor 19	AGG08879.1	*Spodoptera litura*	0.00E+00	90
OR36	422	5	Y	Putative olfactory receptor 44	AGG08877.1	*Spodoptera litura*	0.00E+00	97
OR37	241	–	N	Putative odorant receptor OR20	AOE48025.1	*Athetis lepigone*	7.00E−128	70
OR38	157	–	N	Olfactory receptor 15	JAV45850.1	*Mythimna separata*	3.00E−91	94
OR39	335	–	N	Odorant receptor 17	ALM26206.1	*Athetis dissimilis*	1.00E−173	74
OR40	463	5	Y	Odorant receptor 4-like	XP_011559211.1	*Plutella xylostella*	0.00E+00	75
OR41	95	–	N	Putative chemosensory receptor 10	CAG38111.1	*Heliothis virescens*	2.00E−94	97
OR42	109	–	N	Olfactory receptor 10	JAV45855.1	*Mythimna separata*	5.00E−41	62
OR43	258	–	N	Odorant receptor 62	ALM26245.1	*Athetis dissimilis*	0.00E+00	85
OR44	416	6	Y	Odorant receptor	AIG51890.1	*Helicoverpa armigera*	0.00E+00	74
OR45	161	–	N	Odorant receptor 85	ALM26250.1	*Athetis dissimilis*	2.00E−85	77
OR46	390	6	Y	Odorant receptor	AIG51903.1	*Helicoverpa armigera*	6.00E−169	61
OR47	321	–	N	Putative chemosensory receptor 3	CAD31852.1	*Heliothis virescens*	3.00E−165	79
OR48	407	6	Y	Odorant receptor	AIG51860.1	*Helicoverpa armigera*	0.00E+00	69
OR49	309	–	N	Putative odorant receptor OR9	AOE48014.1	*Athetis lepigone*	2.00E−126	55
OR50	124	–	N	Olfactory receptor 7	JAV45858.1	*Mythimna separata*	2.00E−72	79
OR51	194	–	N	Putative odorant receptor OR36	AOE48041.1	*Athetis lepigone*	2.00E−107	78
OR52	392	5	Y	Putative odorant receptor OR53	AOE48058.1	*Athetis lepigone*	0.00E+00	80
OR53	396	6	Y	Odorant receptor	AIG51856.1	*Helicoverpa armigera*	2.00E−174	60
OR54	89	–	N	Odorant receptor 41	ALM26231.1	*Athetis dissimilis*	3.00E−119	85
OR55	380	4	Y	Putative odorant receptor OR55	AOE48060.1	*Athetis dissimilis*	0.00E+00	66
OR56	70	–	N	Olfactory receptor	KOB68320.1	*Operophtera brumata*	2.00E−21	59
OR57	396	5	Y	Odorant receptor 47	ALM26237.1	*Athetis dissimilis*	6.00E−162	58
OR58	393	5	Y	Olfactory receptor 37	JAV45828.1	*Mythimna separata*	0.00E+00	83
OR59	341	–	N	Odorant receptor 6	AGH58119.1	*Spodoptera exigua*	0.00E+00	77
OR60	133	–	N	Odorant receptor	AIG51873.1	*Helicoverpa armigera*	3.00E−156	74
OR61	408	4	Y	Odorant receptor	AIG51891.1	*Helicoverpa armigera*	0.00E+00	81
OR62	398	3	Y	Odorant receptor	AFC36918.1	*Spodoptera exigua*	0.00E+00	99
OR63	257	–	N	Putative odorant receptor OR60	AOE48065.1	*Athetis lepigone*	2.00E−87	77
**IONOTROPIC RECEPTOR (IR)**								
IR1	329	–	N	Putative chemosensory ionotropic receptor IR68a	ADR64682.1	*Spodoptera littoralis*	0.00E+00	93
IR2	542	3	Y	Putative chemosensory ionotropic receptor IR76b	ADR64687.1	*Spodoptera littoralis*	0.00E+00	94
IR3	722	–	N	Ionotropic receptor 8a	BAR64796.1	*Ostrinia furnacalis*	0.00E+00	79
IR4	874	3	Y	Ionotropic receptor 93a	BAR64811.1	*Ostrinia furnacalis*	0.00E+00	78
IR5	653	3	Y	Putative ionotropic receptor IR1.2	AOE48004.1	*Athetis lepigone*	0.00E+00	69
IR6	539	–	N	Putative chemosensory ionotropic receptor IR1	ADR64688.1	*Spodoptera littoralis*	0.00E+00	77
IR7	269	–	N	Putative chemosensory ionotropic receptor IR87a	ADR64689.1	*Spodoptera littoralis*	0.00E+00	97
IR8	595	3	Y	Ionotropic receptor 7d.3	AJD81625.1	*Helicoverpa assulta*	0.00E+00	80
IR9	606	3	Y	Putative chemosensory ionotropic receptor IR41a	ADR64681.1	*Spodoptera littoralis*	0.00E+00	91
IR10	851	–	N	Putative chemosensory ionotropic receptor IR21a	ADR64678.1	*Spodoptera littoralis*	0.00E+00	92
IR11	630	4	Y	Putative chemosensory ionotropic receptor IR75q.2	ADR64685.1	*Spodoptera littoralis*	0.00E+00	92
IR12	206	–	N	Ionotropic receptor 60a	AIG51919.1	*Helicoverpa armigera*	4.00E−94	71
IR13	459	–	N	Putative chemosensory ionotropic receptor IR75p	ADR64684.1	*Spodoptera littoralis*	0.00E+00	94
IR14	172	–	N	Ionotropic receptor IR64a	AIG51920.1	*Helicoverpa armigera*	5.00E−76	68
IR15	918	3	Y	Ionotropic receptor 25a	AJD81628.1	*Helicoverpa assulta*	0.00E+00	97
IR16	596	–	N	Putative ionotropic receptor IR2	AOE48001.1	*Athetis lepigone*	0.00E+00	82
IR17	361	–	N	Ionotropic receptor 75q.1	AJD81638.1	*Helicoverpa assulta*	1.00E−179	75
IR18	217	–	N	Putative ionotropic receptor IR7d.2	AOE47993.1	*Athetis lepigone*	2.00E−122	75
IR19	343	–	N	Ionotropic receptor IR75p.1	AIG51922.1	*Helicoverpa armigera*	0.00E+00	92
IR20	175	–	N	Putative ionotropic receptor IR75d	AOE47996.1	*Athetis lepigone*	5.00E−76	85
IR21	523	–	N	Ionotropic receptor 2	AJD81622.1	*Helicoverpa assulta*	0.00E+00	68
IR22	364	–	N	Putative ionotropic receptor IR40a	AOE47989.1	*Athetis lepigone*	0.00E+00	92
**GUSTATORY RECEPTOR (GR)**								
GR1	263	–	N	Gustatory receptor 30	KOB69617.1	*Operophtera brumata*	4.00E−14	26
GR2	140	–	N	Gustatory receptor 27	DAA06383.1	*Bombyx mori*	6.00E−12	32
GR3	207	–	N	Gustatory receptor 58	DAA06392.1	*Bombyx mori*	2.00E−18	26
GR4	152	–	N	Gustatory receptor	AIG51914.1	*Helicoverpa armigera*	1.00E−94	87
GR5	199	–	N	Gustatory receptor 62	DAA06394.1	*Bombyx mori*	2.00E−14	28
GR6	151	–	N	Gustatory receptor 58	DAA06392.1	*Bombyx mori*	4.00E−05	47
GR7	379	7	Y	Gustatory receptor 11	DAA06375.1	*Bombyx mori*	1.00E−57	33
GR8	131	–	N	Gustatory receptor 7	DAA06374.1	*Bombyx mori*	9.00E−18	59
GR9	230	–	N	Gustatory receptor 12	AJD81605.1	*Helicoverpa assulta*	4.00E−11	29
GR10	444	7	Y	Gustatory receptor	AIG51908.1	*Helicoverpa armigera*	0.00E+00	95
GR11	199	–	N	Gustatory receptor 62	DAA06394.1	*Bombyx mori*	5.00E−16	29
GR12	446	7	Y	Gustatory receptor 1	AGK90010.1	*Helicoverpa armigera*	0.00E+00	90
GR13	464	7	Y	Gustatory receptor	AIG51907.1	*Helicoverpa armigera*	0.00E+00	96
GR14	411	7	Y	Gustatory Receptor	JAI18131.1	*Epiphyas postvittana*	3.00E−41	35
GR15	377	6	Y	Gustatory receptor 60	NP_001124347.1	*Bombyx mori*	2.00E−12	25
GR16	188	–	N	Gustatory receptor	AIG51910.1	*Helicoverpa armigera*	7.00E−121	88
GR17	180	–	N	Gustatory receptor 8	ALM26257.1	*Athetis dissimilis*	7.00E−62	65
GR18	339	3	Y	Gustatory receptor 12	AJD81605.1	*Helicoverpa assulta*	2.00E−13	28
GR19	160	–	N	Gustatory receptor for bitter taste 93a	XP_012550565.1	*Bombyx mori*	1.00E−68	66
GR20	413	7	Y	Gustatory receptor 53	KOB74473.1	*Operophtera brumata*	1.00E−121	48
GR21	200	–	N	Gustatory receptor 60	NP_001124347.1	*Bombyx mori*	9.00E−13	31
GR22	275	–	N	Gustatory receptor 50	DAA06387.1	*Bombyx mori*	5.00E−90	49
GR23	239	–	N	Gustatory receptor 53	DAA06389.1	*Bombyx mori*	7.00E−64	52
GR24	136	–	N	Gustatory receptor	AOG12970.1	*Eogystia hippophaecolus*	6.00E−22	81
GR25	475	8	Y	Gustatory receptor	AIG51909.1	*Helicoverpa armigera*	0.00E+00	91
GR26	364	7	Y	Gustatory receptor 53	KOB74473.1	*Operophtera brumata*	4.00E−115	51
GR27	476	7	Y	Gustatory receptor	AIG51911.1	*Helicoverpa armigera*	0.00E+00	80
GR28	258	–	N	Gustatory receptor 53	KOB74473.1	*Operophtera brumata*	1.00E−81	54
GR29	503	7	Y	Gustatory receptor	AGA04648.1	*Helicoverpa armigera*	0.00E+00	94
GR30	341	–	N	Gustatory receptor 7	ALM26256.1	*Athetis dissimilis*	0.00E+00	79

*TMD, transmembrane domain*.

### OBPs

**Figure 5:**
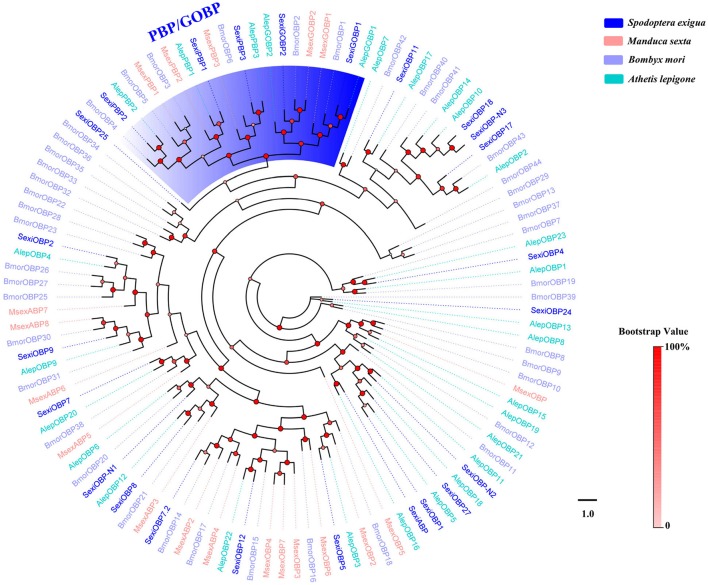
Phylogenetic tree of insect OBP. The *S. exigua* translated genes are shown in blue. Amino acid sequences used for the tree are given in Table S1. This tree was constructed using PhyML based on alignment results of ClustalX.

We obtained a complete set of 24 different unigenes encoding putative OBPs in *S. exigua* (Table 2), of which 3 were newly identified. Sequence analysis revealed that 23 sequences were predicted to have full-length open reading frames (ORFs) and encoded 118–239 amino acids, but only 3 of the 23 SexiOBPs did not have signal peptide sequences (Table 2). The phylogenetic analysis showed that all 24 SexiOBPs were clustered in an OBP tree with *Manduca sexta, B. mori*, and *Athetis lepigone* (Figure 5), including 5 SexiOBPs (SexiPBP1-3, SexiGOBP1-2) clustered into the PBP/GOBP subfamily. The results suggest that these SexiOBPs belonged to the insect OBP family and should have the corresponding functions of the insect OBP (Poivet et al., 2012; Jeong et al., 2013; Pelosi et al., 2014; Liu et al., 2015a). The two new SexiOBPs (SexiOBP-N1 and SexiOBP-N3) encoded protein with high identities (97 and 99%) to OBPs in *Spodoptera litura*, respectively, indicating that SexiOBP-N1 and SexiOBP-N3 might have conserved functions in the two closely related species, such as recognizing the same host plant volatiles (Li et al., 2013; Gu et al., 2015). Therefore, they can be considered as target genes to simultaneously prevent and control these two pests (*S. exigua* and *S. litura*) in the future.

### CSPs

**Figure 6:**
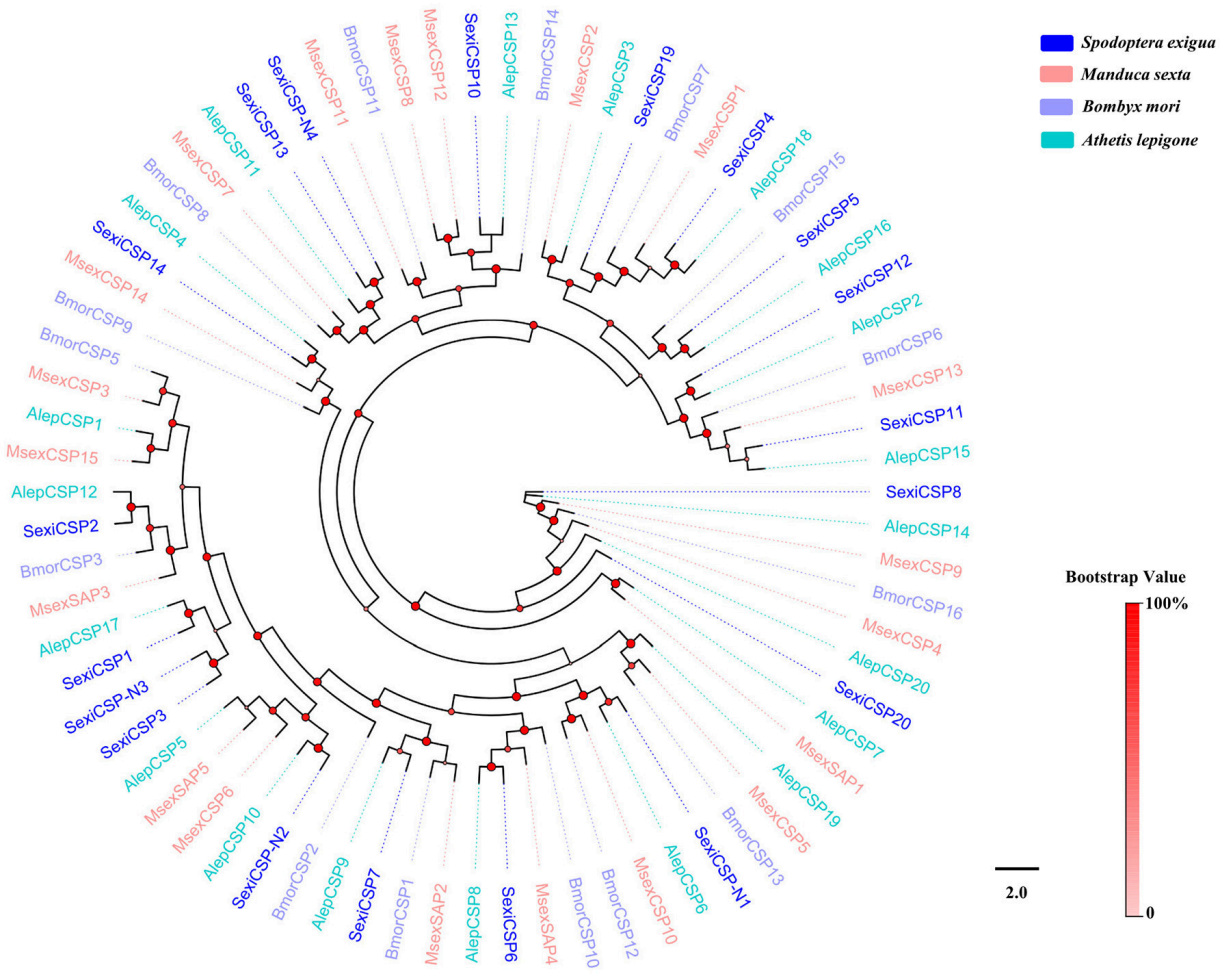
Phylogenetic tree of insect CSP. The *S. exigua* translated genes are shown in blue. Amino acid sequences used for the tree are given in Table S1. This tree was constructed using PhyML based on alignment results of ClustalX.

Nineteen putative genes encoding CSPs were acquired in *S. exigua* based on the analysis results from the transcriptomes of the six chemosensory organs, of which four were newly attained (Table 2). Among the 19 SexiCSPs, 18 had full length ORFs with 4 conserved cysteines in the corresponding position and a predicted signal peptide at the N-terminus. The constructed insect CSP tree using protein sequences from *S. exigua, M. sexta, B. mori*, and *A. lepigone* (Figure 6) indicated that all 19 SexiCSPs were distributed along various branches and each clustered with at least 1 other moth ortholog. Thus, we inferred that these SexiCSPs should have a similar chemosensory function in insects, especially moths (Lartigue et al., 2002; Campanacci et al., 2003; Zhang et al., 2014). Similar to SexiOBPs, we also found three of the four new SexiCSPs (SexiCSP-N2, SexiCSP-N3, and SexiCSP-N4) encoded proteins with high identities (99 and 100%) to CSPs in *S. litura*. This showed that they were very similar, maybe even the same CSPs, and might play the same role as OBPs in the two moths. In future studies, we intend to use the combination of *in vitro* (Jin et al., 2014; Zhang et al., 2014) and *in vivo* (Zhu et al., 2016; Dong et al., 2017; Ye et al., 2017) methods to explore the exact function of these conserved OBPs and CSPs in the two closely related species. In addition, we plan to study the exact functions of all the unknown functional OBPs and CSPs of *S. exigua*, which will help us define the odorant binding spectrum of each gene. This will provide potential behavioral disturbance agents to control the moths by using reverse chemical ecology methods (Zhu et al., 2017).

### ORs

**Figure 7:**
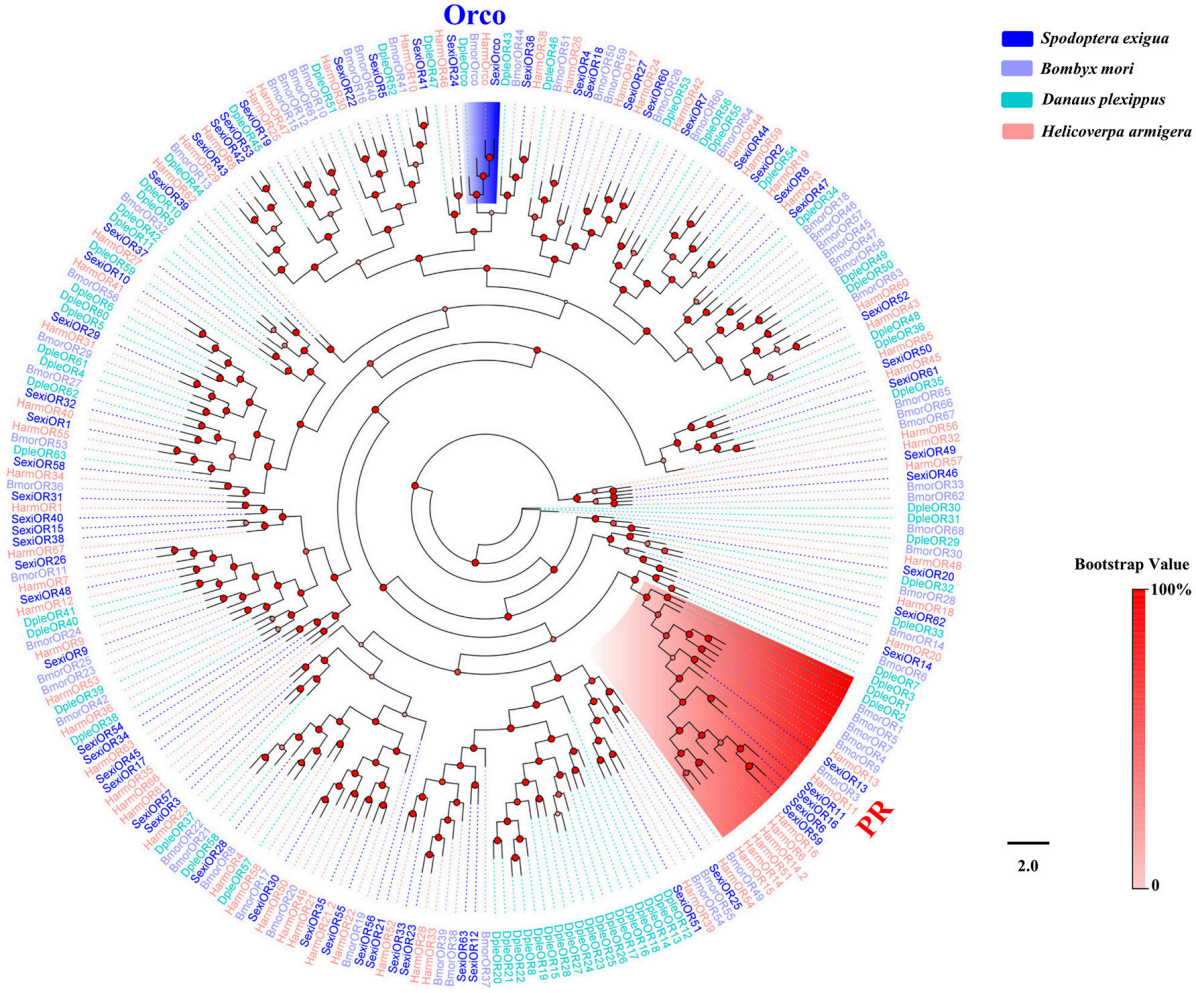
Phylogenetic tree of insect OR. The *S. exigua* translated genes are shown in blue. Amino acid sequences used for the tree are given in Table S1. This tree was constructed using PhyML based on alignment results of ClustalX.

Sixty-four different unigenes encoding putative ORs were identified by analyzing the transcriptome data of *S. exigua*, of which 57 were newly obtained (Table 3). A total of 28 out of 64 SexiORs contained full-length ORFs that encoded 351 to 473 amino acids with various transmembrane domains (TMD). The phylogenetic analysis showed that all 64 SexiORs were clustered in an OR tree with *B. mori, D. plexippus*, and *H. armigera*, with each clustering having at least one other moth ortholog (Figure 7). In accordance with previous studies (Liu et al., 2013), we also identified a chaperone and higher conserved insect OR—SexiOrco (Krieger et al., 2005; Nakagawa et al., 2005; Xu and Leal, 2013; Missbach et al., 2014) and four pheromone receptors (SexiOR6, 11, 13, and 16) (Table 3, Figure 7), which suggests that our sequencing and analysis methods were reliable. The results of the phylogenetic and sequence homology analyses showed that we were able to obtain the fifth PR gene of *S. exigua*, SexiOR59. Liu's research (Liu et al., 2013) found that only two PRs (SexiOR13 and SexiOR16) showed higher electrophysiological responses to the three sex pheromone components (Z9, E12-14:OAc, Z9-14:OAc, and Z9-14:OH) of *S. exigua*; however, no PRs displayed specific or higher response to the fourth pheromone component Z9, E12-14:OH. Therefore, further studies are required to determine whether SexiOR59 can respond highly or not to Z9, E12-14:OH or other pheromone components. Additionally, other researchers have found that several non-PR ORs could respond to host plant volatiles, such as SlitOR12 of *S. litura* (Zhang et al., 2015c), EpstOR1, and three from *Epiphyas postvittana* (Jordan et al., 2009). Therefore, some ORs of the 58 non-PR ORs in *S. exigua* might play a similar role in the chemosensation of the volatiles in host plants.

### IRs

**Figure 8:**
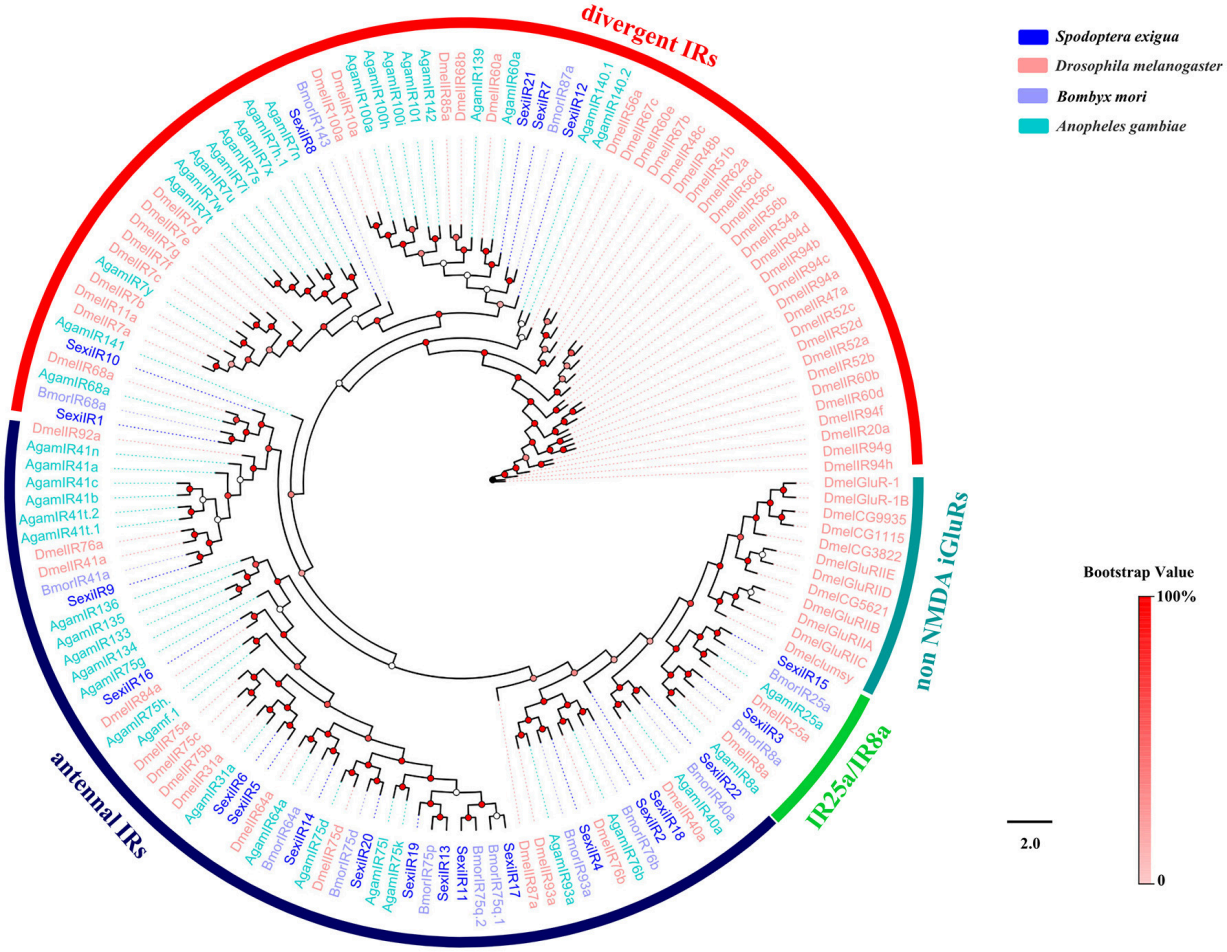
Phylogenetic tree of insect IR. The *S. exigua* translated genes are shown in blue. Amino acid sequences used for the tree are given in Table S1. This tree was constructed using PhyML based on alignment results of ClustalX.

A total of 22 putative IR genes in *S. exigua* were identified, of which 16 were newly obtained (Table 3), and the SexiIRs number was similar to several other insects (Croset et al., 2010; Liu et al., 2014b; Xu et al., 2015). Only 7 of these genes had a full-length ORF (SexiIR2, 4, 5, 8, 9, 11, and 15) that encoded 542 to 918 amino acids with 3 or 4 TMD. We then constructed an insect IR tree using protein sequences from *S. exigua, Drosophila melanogaster, B. mori*, and *Anopheles gambiae*, which indicated that all 22 SexiIRs were clustered into 3 subfamilies of insect IR: 14 antennal IRs (SexiIR2, 4-6, 9, 11, 13, 14, 16-20, and 22), 6 divergent IRs (SexiIR1, 7, 8, 10, 12, and 21), and 2 IR25a/IR8a (SexiIR15 and 3), but no SexiIRs belonged to non-NMDA IGluRs subfamilies (Figure 8). This is similar to the conserved co-receptor Orco, where IR25a and IR8a of the insect were also co-receptors and could be co-expressed along with other IRs to ensure that insects could accurately detect external odorants via chemosensory organs (Abuin et al., 2011). Therefore, the co-receptors SexiIR15 (25a) and SexiIR3 (8a) might play the role of molecular chaperone to help with other SexiIRs functions.

### GRs

We first identified 30 different unigenes encoding putative SexiGRs in the present study (Table 3). Sequence analysis revealed that 12 sequences were predicted to have full-length ORFs that encoded 339–503 amino acids with 3–8 TMD. This number of SexiGRs is higher than that of other species based on the transcriptome analysis, such as *H. armigera* (10 GRs) (Liu et al., 2014b), *H. assulta* (18 GRs) (Xu et al., 2015) and *Hyphantria cunea* (9 GRs) (Zhang et al., 2016), but lower than that of 3 species whose genomes have been sequenced, *B. mori* (69 GRs) (Wanner and Robertson, 2008; Sato et al., 2011), *D. plexippus* (58 GRs) (Zhan et al., 2011; Briscoe et al., 2013), and *Heliconius melpomene* (73 GRs) (Briscoe et al., 2013). This suggests that there is a high chance of identifying more SexiGR genes when the genome of *S. exigua* is successfully sequenced in the future.

**Figure 9:**
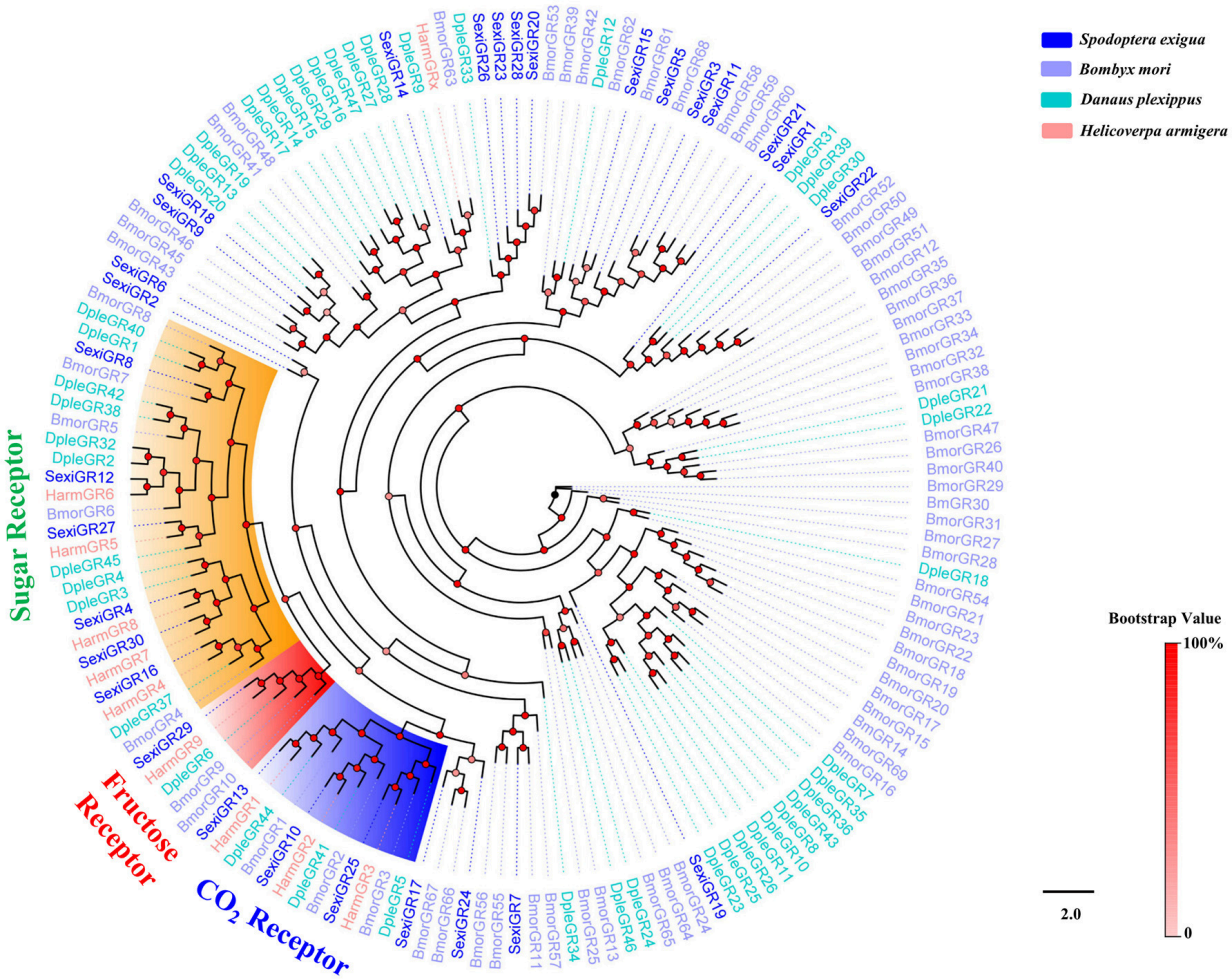
Phylogenetic tree of insect GR. The *S. exigua* translated genes are shown in blue. Amino acid sequences used for the tree are given in Table S1. This tree was constructed using PhyML based on alignment results of ClustalX.

An insect GR tree using protein sequences from *S. exigua, B. mori, D. plexippus*, and *H. armigera* was then constructed, and the tree showed that 3 SexiGRs (Sexi10, 13, and 25) were clustered in the CO_2_ Receptors subfamily, 6 SexiGRs (SexiGR4, 8, 12, 16, 27, and 30) were clustered in the Sugar Receptor subfamily, and 2 SexiGRs (SexiGR13 and 29) were clustered in the Fructose Receptor subfamily (Figure 9), indicating that these SexiGRs might be involved in the detection of CO_2_ (Jones et al., 2007; Kwon et al., 2007), sugar (Sato et al., 2011), and fructose (Jiang et al., 2015; Mang et al., 2016). Other SexiGRs, which do not belong to the three subfamilies, might be involved in other taste perception processes.

**Figure 10:**
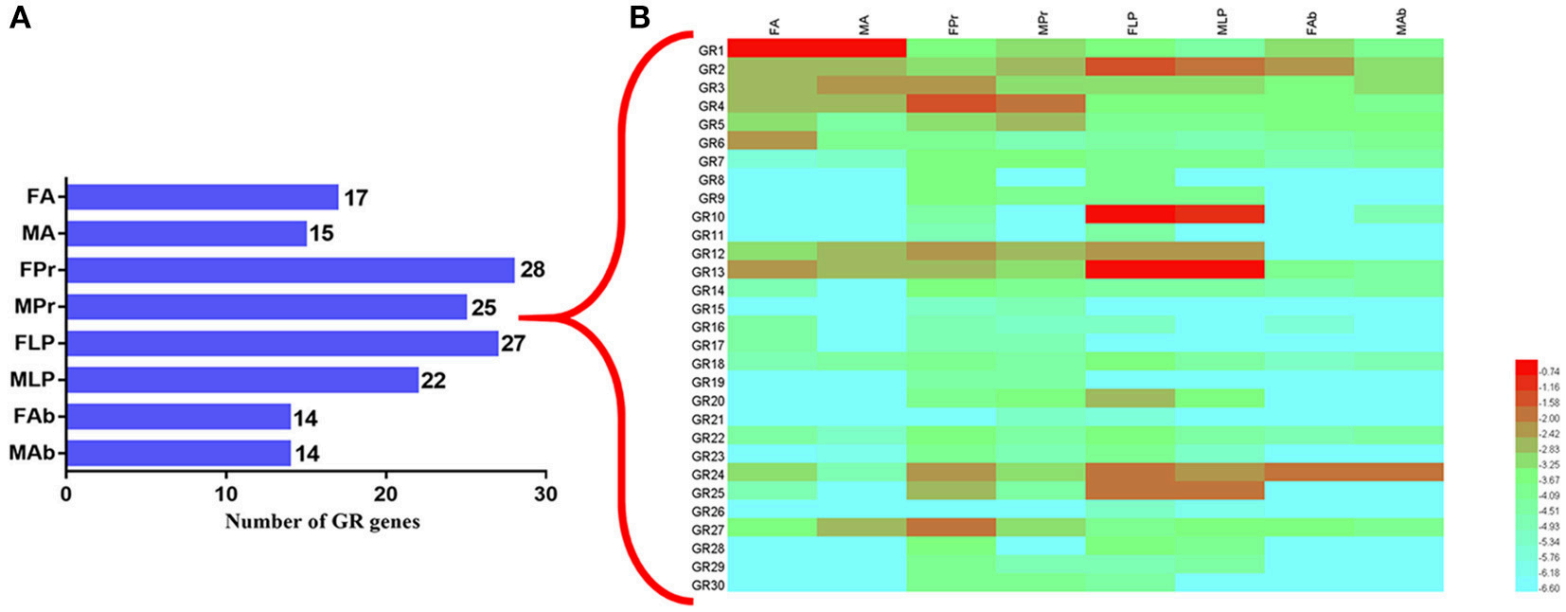
Expression pattern of the *SexiGRs*. **(A)** The number of GR genes expressed in different organs of *S. exigua*. The digits of the histogram represent number of GRs. **(B)** relative expression levels of *SexiGRs* using qPCR. FA, female antennae; MA, male antennae; FPr, female proboscises; MPr, male proboscises; FLP, female labial palps; MLP, male labial palps; FAb, female abdomen; MAb, male abdomen.

To better infer the potential functions of these SexiGRs, we applied the qPCR method to investigate the expression profiles of all SexiGRs in six chemosensory organs (FA, MA, FPr, MPr, FLP, and MLP) and two non-chemosensory organs (Female abdomen, FAb and Male abdomen, MAb) (Figure 10). The results showed that the organ with the highest SexiGRs expression was FPr (28 genes), followed by MPr (27 genes), FLP (25 genes), and MLP (22 genes), indicating that SexiGRs mainly exist within the gustatory organs, not the olfactory or non-chemosensory organs. This explains why the numbers of GR based on the antennae or non-gustatory organs transcriptome of other insects (Liu et al., 2014b; Xu et al., 2015) are lower than the SexiGRs in the present study. Additionally, we found 4, 16, 11, and 1 SexiGR genes that were highly expressed in the antennae, proboscises, labial palps, and abdomen of *S. exigua*, respectively, and some genes also showed differences in sex expression, which suggests that SexiGRs not only plays a pivotal role in gustatory processes (Jiang et al., 2015; Poudel et al., 2015), but might also be involved in olfactory (Agnihotri et al., 2016; Poudel et al., 2017) and other physiology processes (Xu et al., 2012; Ni et al., 2013). These results indicate that the proboscises and labial palps play more important roles in the taste perception process of than the olfactory organs do, which provides an important reference for future study of the taste perception mechanism in *S. exigua* as well as in other moths.

In conclusion, 159 genes encoding putative chemosensory genes were obtained by analyzing six chemosensory organs of *S. exigua*. Our approach proved to be highly effective for the identification of chemosensory genes in *S. exigua*, for which genomic data are currently unavailable. As the first step toward understanding gene functions, we conducted a comprehensive phylogenetic analysis of these genes and investigated all SexiGRs expression patterns, most of which were highly expressed in gustatory organs. The present study greatly improves the gene inventory for *S. exigua* and provides a foundation for future functional analyses of these crucial genes.

## Author contributions

Y-NZ conceived and designed the experimental plan. Y-NZ, J-LQ, M-YL, and X-XX performed the experiment. Y-NZ, X-YZ, TX, and LS processed and analyzed the experiment data. J-WX and C-XL provided important suggestions to help modify the manuscript. Y-NZ wrote the manuscript.

### Conflict of interest statement

The authors declare that the research was conducted in the absence of any commercial or financial relationships that could be construed as a potential conflict of interest.
