# Supplementary material for: Identification of Chemosensory Genes Based on the Transcriptomic Analysis of Six Different Chemosensory Organs in Spodoptera exigua
Source: Front Physiol. 2018 Apr 24;9:432. doi: 10.3389/fphys.2018.00432 (PMC5928209; doi:10.3389/fphys.2018.00432)
Supplement: Supplementary file 1 [file Table_1.DOCX]

**Table S1 Amino acid sequences of *S. exigua* used in phylogenetic analyses.**

**OBP**

>SexiPBP1

SQELMMKMTKGFTKVVDECKAELNAGEHIMQDMYNYWREDYQLINRDLGCMILCMAKKLDLMEDQKMHHGKTEEFAKSHGADDEVAKKLVSIIHECEQQHAGIADDCMRVLEISKCFRTKIHELKWAPNMEVIMEEVMTAV

>SexiPBP2

SQDVMKNLAINFAKPLDDCKKEMDLPDSVTTDFYNFWKEGYELTNRQTGCAILCLSSKLEILDQELNLHHGRAQEFAMKHGADETMAKQIVDMIHTCAQSTPDVAADPCMKTLNVAKCFKLKIHELNWAPSMELIVGEVLAEV

>SexiPBP3

SKDAMKYITSGFVKVLEECKQELNMNDHIIADLFHFWKLEYALLSRDTGCVIICMSKKLDLLDANGRMHHGNAQEFAKRHGAGDDVASKIVQIIHDCEKKHERDDDECLRVLEVAKCFRTGIHDLDWQPKVEVIVSEVLTEI

>SexiGOBP1

DVNVMKDVTLGFGQALDKCRQESQLTEEKMEEFFHFWRDDFKFEHRELGCAIQCMSRHYNQLTDSSRMHHDNTEQFIKSFPNGEVLARQMVELIHSCEKQYDHEDDHCWRILHVADCFKQGCVQRGIAPSMEMMMTEFIMEAEAR

>SexiGOBP2

EVMGTAEVMSHVTAHFGKALEECREESGLFAEVLEEFQHFWREDFEVVHRELGCAIICMSNKFSLLQDDTRMHHVNMHDYVKGFPNGHVLSEKLVELIHNCEKQFDSMTDDCERVVKVAACFKVDAKAAGIAPEVAMIEAVMEKY

>SexiOBP1

TAEEKAALIEAVKPYIQECSKEHGVTPEDIKSAKEAGNADGINACFLRCVYNKAGVINDKGEYDADKALEKLKKFVSNEDDYAKFAEIGKKCASVTETSVSDGEAGCERAALLTSCFLEHKSEVHA

>SexiOBP2

NEKGNKLDRPFASECIKETGVKNELLEEAKKGIISEDPAFKAFTYCFFKKIGIVGEDGLLNRDVAIAKLPSGVDKSEAEKLLDSCKSKTGKDAVDTVFEIFKCYQQGTKSHIMFAS

>SexiOBP4

LTEEELKMEFTKLIMKCNKDGKVDMTELVQLQNYVVPTKQTTKCVLACAYKAAEVMNAKGEYDIDHAYKVAEMMKNGDEKRLVNAKKMADLCVKVNEQSVSDGEKGCDRAAMIFKCTVENAPKFGFKL

>SexiOBP5

MTMKQIRNTGKMMRKTCQPKNNVEDEKIDPIAEGVFIDEKEVKCYMACIMKMANTIKNGKLNYDAAIKQADLLLPDDIKEPAKEAITACKKVADAHKDICDASFHITKCIYNHNPGIFYSP

>SexiOBP7

ASTKEAMTTTMTDQVNSIEVDVLAVMDMCNDSYRIDPTYLQALNESGSFIDETDKTPKCFIRCVFENVGIVSEDGMQLNPARAAVIFAGERNGKPMEDIADMTALCATDRQETCPCDRSYKFLRCLMSMEIERYEKS

>SexiOBP7.2

MSRQQLKNSGKMLKKNCMNKIGVTEDQVGSIDKGKFIEDRKVMCYIACIYELTNVIKNNKLNYEASIKQIDLMYPPDIKESAKAAVEKCKDVQKKYKDICEVSFYAAKCMYEFKPEDFIFA

>SexiOBP8

GKDKPVFSDEIKEIIQTVHDECVAKTGVAEEDITNCENGIFKEDAKLKCYMFCLLEEASLVDDDDTVDYDMLVSLIPDEYYERTTKMIFACKHLDTPDKDRCQRAFEVHKCSYEKDPDLYFLF

>SexiOBP9

LTDEQKEKLKKHRTECLSETKVDEQLVNKLKGGDYKTESEPLKKYALCMMMKSELMTKEGKFKKDVALAKVPNPADKPTVEKLIDACLANKGNTPHQTAWNYVKCYHEKDPKHAIFL

>SexiOBP11

MKEGNRYSHERRITNDSGDQLMVINATDDDYSGYGSGNMGEKLLTSVPRPASSSNNINKNNTRRTRRNEPFLNRPDSDQCLSQCVFANLQVVDSRGIPREAELWNKVQTSVTSQQSRSALHDQIRACFQELQSEAEDNGCSYFNKLERCLMLRFSDRKVDGKGNPKKSSTEQT

>SexiOBP12

MTREQIKNSGKLIKKTCSAKNDLTEDEVKDVDKGKFIEKKDFMCYIACVYKMGQSVKGSTINHDMMLRQVDMMFPNDMKAPVKAAIEHCRPVAKNYKDFCEASYWTAKCIYDFDPANFMFP

>SexiOBP17

DVVETPTKREIKASLKPLSVCCDIPELADDRQLVKCSNPKPPGPCEDVQCIFEVSGFLTDKNTLNKAAYRSHLQKWEKNHPGWTDAIYKAITDCVDNDPRQHLDVACKAYDVFTCTGIAMLKKCPEAAWKC

>SexiOBP18

APNKASGTFCGLTPNNMFKCLNNPRVLNLEAAAKCTSQVTECEKITCVFREQKWSKHGVIDKAKIRAHFEQYETEHPEWAPAVQHVKAFCLAPELRAQGVFLNCPAYDIMQCALASFIKHASPSVWSTEQNCDYPKAYAADCPVCPSDCYSAAIPIGSCNACYLQPRTV

>SexiOBP24

DSAISADSESRCRNPPTAPQKIERVITLCQDEIKLSILREALDVIKEEHTMPAQRRRDKREVPFTHDEKRIAGCLLQCVYRKVKAVDGYGFPTLEGLVGLYSDGVNERGYFMAVLEASRECLMKNHDKFSRTVPMDNGRNCDVSFDIFECISDRIGEYCGTSGL

>SexiOBP25

DPVPFITKCKWDDTKCNKESSQAVVGIFSAGLPEYNVEKSDPLQIDYVDASSPNMKLIVTDVVVTGLKNCEVKKMQRFEDSSKLIVKILCSTELKGKYDMKGQLFVIPIEGKGDLYANVPKIQINAEVDLNIKKGKDGKDRWLVKSWRHTFDLKDKSTVKFENLFPDNEFLRTSTNELIAQNGNDVIIEIGANLIKALVGKVVENIKKFFLAVPIEDLSL

>SexiOBP27

MSAMKPIVDECAKKHGVTLEALLAAKASGKIDGIEPCFYSCVYKKTEFLNSKGEYDVDNSLVKLKKYISSDDDYAKFSQIGKDCASVNSKSVGDGEAGCERAVLLTQCFLEHKGAVPM

>SexiABP

MSGDEEAGVRDALRPYVQECADEYGITEEQFEEAKKKASADDIDPCFMSCFLKKAEFFDAQGKFDVDSTMAFAKEHLSSEPAMKFVEAVGDECVKINDEDVSDGDKGCDRAKLLFDCIAETKKKMD

>SexiOBP-N1

AVEMDEDMAELARMVRENCAGETGVDVALVEQVNAGAELMPDDKLKCYIKCTMETAGMMADGEVDIEAVLALLPPSLAEHNAPALRACGTQRGADHCDTAFRTQQCWQNANRADYFLI

>SexiOBP-N2

MSECADKFGVTEHDYRTALISGDVDAIDPCFWRCYFKNMGVFNAEGLYDLEATLHFIKTTIHDDYTQVQKIATLCEKVNNEVVNKGEAECVRAALLMTCLLEGGGDRSLH

>SexiOBP-N3

APNSSPGTPNANPGTYCGVTPDNIYRCLNNPRVVTPEVSTKCGSQFTECEKMTCIFRELKWSKRGAIDKAKVRAYFDQYETEHPEWAQAVQHVKAFCLASELRAQGVFLNCPAYDIMQCVLASFI

**CSP**

>SexiCSP1

RPDGSTYTDRYDNINLDEILGNRRLLTPYIKCILEEGKCTPDGKELKSHIREALEQNCAKCTDAQRNGTRRVLGHLINNEEESWNRLKAKYDPQSKYTVKYELELRKLKQ

>SexiCSP2

RPDDSHYTDRYDNVDLDEILSNRRLLVPYIKCILDQGKCAPDAKELKEHIREALENECGKCTETQKKGTRRVIEYLINNEEEYWNELTAKYDPERKYTTKYEKELKKIKA

>SexiCSP3

HPHDSHYTDKYDNIDLDEILNNKKILTSYINCCLDLGKCTPDGKELKSHIREALENKCGKCTEAQKNGTRKVMTHLINFEPDYWNQLCAKYDPEGKYKAMYEKEYKTLVH

>SexiCSP4

EDKYSTENDDLDIEAVVADLDTLKGFVGCFMDAMTCHAVAADFKKDIPDAVATSCAKCTNAQKHIFHKFLLGLKEKLPSDYEAFKKKFDPQGQYFEALEAAVASS

>SexiCSP5

QEKFYDRRYDYYEIDTLIQNPRLLKKYLDCFLGKGPCTPIGRVFRQILPEAVQTACKKCTPSQRRLARKTFNAFKGYFPETHEELRKKLDPKNKYYEAFEKAISSA

>SexiCSP6

KPASTYTDKWDNINVDEILESQRLLKAYVDCLLDRGRCTPDGKALKETLPDALEHECSKCTEKQKKSSDKVIRHLVNKRPDLWQELSGKYDPENIYQERYKNQLDAVKRQ

>SexiCSP7

DDEKYTSKYDNIDLDEILTNKRLLTAYVNCIMERGKCSPEGKELKEHLVDAIETGCTKCTENQEKGAYKVIEHLIKNELDIWRELTGKYDPSGKWRKTYEDRAKANGIIIPE

>SexiCSP8

LHVQAGPQMTDAQLEQTLADKSTMQRHIKCALGEGPCDPVGRRLRTLAPLVLRGACPQCSMQETRQIRRTLAFVQRNYPWEWAKIVRQYG

>SexiCSP10

QANRYENFNPDAIVQNDRILLAYYKCVMDKGPCTRDGKNFKRVLPETLATACGRCNPKQKTIVRKLLLGIRSKSEPRFLELLDKYNPDRSNRDALYAFLVTGA

>SexiCSP11

ADKYNPKYDNFDVDTLISNDRLLKAYINCFLEKGRCTPEGSDFKKALPEAIETTCAKCTDKQKGNIRKVIKAIQQKHPKEWEDLVKKNDPSGKHRGNFDKFIQGSS

>SexiCSP12

RPDDGFYDKKYDNFNADELIENDRLLKSYAHCFLEDGKCTPEGNDFKKWIPEATTTSCGKCTDKQKVLVAKTIKAIKEKLPAEYEALVKKHDPEHKHHDDLNKFLEKYAP

>SexiCSP13

RPETYDTRYDNFDVEALVGNVRLLTAYGHCFLGTGPCTPEGSDFKKTIPDALRTGCGKCSPKQRHLIRVVVQGFQNKTPALWQQLVKKEDPNGEYKEIFTRFLNAKD

>SexiCSP14

QDVNDMVNMPKYDQRYDYLDVDAIFANKRLVRNYVDCLINAVRCTPEGKALKRILPEALRTKCVRCTERQKRTAVKVIKRLKNEYPDEWSKLASRWDPTGDFTRYFEEFLAKEHYNTIPGSGSALPTSAPIAPPRVSPLPPSTTPTPGPTESTPPRPLVLNRFGDDGELMMGSPSSAGVTPRPMTQATTRPSTTTKTPSTRPIPPRPTMMTWAGAASNTQSTRFPLRPVSEISPPYSTAITLIDQIGYKIIKTTELVTDTLRNTVRAVVGR

>SexiCSP19

QETYGTQYDNVNGEAIVSDDQQFQSFVDCFMGAATCNEPAAAFKKVLPEAIVQACAKCNPAQKHLVRVFLEAYSKKMPQEYEKFKDLFDPERKYFPKFEASVAGF

>SexiCSP20

QSQRPPVSDTALDDALQDKRFIQRQLKCALGEGPCDPIGKRLKTLAPLVLRGACPQCTPQETKQIQRTLSYVQRNYPQQWAKIVRQYAG

>SexiCSP-N1

TETSTYTTKYDGIDLDEILSNDRLLTGYVNCLMDIGPCTADGKELKKNLPDAIENDCKKCTERQREGADRVCHYLIDNKPEDWTKLEEKYKSDGSYRAKYLASKQTKDEKESNATKSSEDTNNVSKE

>SexiCSP-N2

ETYTDRYDHINIDEIIENRKLLVPYIKCTLDQGRCTPEGRELKAHIKDAMQTSCSKCTEKQKKGARKVVKHIRAKEQEYWKQILAKYDPEDQYKENYETFLAAED

>SexiCSP-N3

NIDLDEILNNKKILASYVKCCLDQGKCTPDAKELKSHIKEALENRCGKCTPAQKDGTRKVLTHLINHEPEMWNQLCEKYDAEGKYRKMYEDEYKSVKH

>SexiCSP-N4

RPESYDTRYDNFDVEALVGNVRLLTAYGHCFLGNGPCTPEGSAFKKTIPDALRTGCGKFSPKQRHLIRVVVQGFQNKTPALWQDLVKKQDPNGQYKEIFTRFLNGRD

**OR**

>SexiOrco

MMTKVKAQGLVSDLMPNIKLMQAAGHFLFNYHSENGGMTGLLRKIYASTHAILITIHFACMGINMAQYSDEVNELTANTITVLFFTHTIIKLGFFALNSKSFYRTLAVWNQSNSHPLFTESDARYHQIALTKMRRLLYFICGMTVLSVVCWVALTFFGESVRLITSKETNETLTEVAPRLPLKAWYPFNAMSGTMYIIAFAFQVYWLLFSMAIASLMDVMFCSWLIFACEQLQHLKAIMKPLMELSASLDTYRPNTAELFRASSTEKSEKIPDTVDMDIRGIYSTQQDFGMTLRGAGGRLQNFGQQNNNPNGLTPKQEMLARSAIKYWVERHKHVVRLVASIGDTYGTALLFHMLVSTITLTLLAYQATKINGINVYAFSTIGYLSYTLGQVFHFCIFGNRLIEESSSVMEAAYSCQWYDGSEEAKTFVQIVCQQCQKAMSISGAKFFTVSLDLFASVLGAVVTYFMVLVQLK

>SexiOR1

MKKGDPKIRQHMKDYTKYTRIITYMFWTMVVITNFLLILTPLLKYVSSISYRESIRKGIEPLPQILCSWFPFDNGRMPGYLISVIVHIIMGSQGSGVLAVYDMNAVAIMSYLKGQMIILREKCDSLFDDVTSAQDVLDRIKECHRHHNTLLKHFTLFNSLLSPTMFVYVLMCSITICGSVVQFSSKEATASQKLWVFQYTTGLISQLFLYCWHSNEVTLHSKLVDRGIYNSDWWKSNVHLRKHLLLLGGKLNQCMILDAGPYTTLSIPTFIEILKGSYSFFTLFSQIQES

>SexiOR2

MGLIKNLCLKLSYTKAIDRSSGKLETIFFENYYRIAYVAGMSTADDDIGYLVYSNVAKLMIVLLVLGVVWYGFMETTSFDEFAANLNVSILQFITFYRYRNMLAHEKFYKELASSMESPYFDISTEQRQKLVEFWSRTNVKYLKLLMGLGNCTLLAWFIFPLVDDIEYNLIVGVYLPFFYKSPSMYPLAYMLSVLFFIYISHFVMVTDLKMQTHLIHLLCQFAVLGDCFENMIPDCMAGFEGVPRNQLMYNNQFAARYTERLGNLVKQHKALLGHAMNLRDTLSGPLLGQLAASGVLICFIGYQATATIGQSVVECMTSFLFLAYNLFDFYMICRWCQEITNQSANVGEAIYCSGWECGVSKLPGVRSTIMFVIARANKPLVLTAGGMYDLSLTSYTSLVKTSYSALTVLLRFRN

>SexiOR3

MDEAFLAFHRVLSFAGISIFAKQNWDSNRWLVHQTFNFIIGVLCFIFTTGFVVTNVSDFLLCIQGACIWTTGVIMTITLGVCLIFRKDFRNFLEEMAFRDHMLEMPLIDHVLLVSSGGEKIQELRDLVTGSQEKLFKYTTILLRVYVASVFLCATLYLCSPVYGMMVREDESLRLLAFDMWFPWSLEDYTVYIISFIFHAYAGYLCCIAYPGLQSIIILLLGQIIRQIRILTFILLHTNELVLEVTRVQDKRWQYMCTLVLSQCVDHYVKIKSFSNRLNVICRPFYLTLILVAIMLVCMCSVKIAVSNKVSLDTMKYYIHEFCFILVVLMFCLVGQQVDNECEALERAVTEKWYIFNHNHKSNIRIFNMALGQRMPIYIFGTITLSLPTFTWFIKTGMSFFSLVMSVLEEETQ

>SexiOR4

MSVLITFQAYGNVTMDCTIVAFYAQAKIQLKMLRYNLEQLVVFTNTKKYSSIKHQYRDQGEEETELQERLKKCVIHYHQIVRFTKEVESIFGEAMVVQFFVMAWVICMTVYKIVGVSLLIQDKAKVPNKI

>SexiOR5

MEAIKNTTLFLGRPKKILTAHGVWPHPNNYIVLRTLYMLFVMWTQYSFLLFEIIYIVNVWGDIDEVSEASYLLFTQASLCYKSTTFMINKKNLLELLDIMDCDIFKPKSAEHVK

>SexiOR6

MGLKKFLFEDECVKGITAPTDYLYVKLLRMSLRIIASWPHKELGEKDPVILNTFLKYFYLAATSACQFGSYLYLRTYNDELTMMEAGHSYLMIMMTFIDISRIVTLTFRKKYRLICKEFFTKMHLFYFKDISEHAMETHKRVHLMSHLFTLWLIFQMVFGVPLFNLIPMYYNYAAGRFKPGGTQNSTFEHSMYYEYPFDTLRDIRGYIVANIINWILSCLCVTWFCMCDLILSLMVFNIWGHLRMLIYTLYHFPRPSIETTITIEGGLTVTSAKYSDEETLEVFKKLKECVNYHRRIVEFNSQVSDVFGPMLVCYYLYHQTSGCLLLLECSQMTAPVLMRYLPLTIVCTQQLIQLSVIFELIGTESEKLLNAVYSVPWECMDTSTRKFVSFFLMNVREPIHVKALGIANVGVTTMAAILRTSLSYFTFLRSI

>SexiOR7

MTETRPKHYFGFHYRILRFLGLGWWHDPEEGNTSNFPGWYLYYSIATQLIWVAGFVGLETIDPFVGEKEMDRFMFSLSFVITHNLTLIKLYIFFFKNADIQEIVHTLEIGIYDYYQNDEKNRKTVRMSKIVTAAFIFFGWVTIGNGNVYGTIQDLKWKSLVATLNDTDVRPVRTLPQPIYIPWDYQKDASYIPTFVLETVGLLWTGHIVMTIDTFIASVILHMGSQFEILHEAITTAYDRTIASLREGVHADANNLREGESSKLSEDENNERIVKSFVPKEDIDAALKNTFRNCFRQHQVLINCVEKFSRTYSYGFMTQLLSSMAAICVVMVQVSQDASSFKSVRLITSVAFFVAMILQLGMQCLTGNELTIQAERISDAIMQCKWERIPPSQRRLLLIMMMRAQRPLRLTAAGFTHMDNACFLAIMKAAYSYYAVLSQKQQ

>SexiOR8

MILNVFFFQDRDDLNKVMDTMFLLLTNSDSIYKQIVLWKKANRIEILLNIMKGNMPSYFI

>SexiOR9

MIEQFEKCLKRVSFFWKLLGMSLDYRGDSKTMTEFIRRHKIYVAQLFSLNCENVTQILWVFEAVITGKTFLEITYLIPCLIHCIIGVFKTVSMLYYAKYNYEFISTIKRLLMNLTPSTEEDNIFMKNLIDKHVLMLMNISKRVVIVVSVGLVMFALGPVFVILPHYFKTNEVKLEMPFIAYYPFNEFDVRIYPFLYVHQLWTALSATLMVYGPDCFFFTCCTFIHIQFAILSYDMERLVNDDSRICDRNRLKELVVRHIELMRCVELVEKVFSKSILFNAMTSSVIICVTGFNVSVSIYLNLCFLFIIKRFS

>SexiOR10

MLKNILQKLENPKRPLLGPNVKVLEFWGLLLPKNVFKKYFYLLMHLMVTIFTATEYIDVWFVKSDLNLLLNNLKITMLATISVIKVSTFLSWQKYWIDIIEYVTRADLDQRKTVDKEKLDLLKAFTQYCRKITYLYWSLMYTTVVIVMVQPIFKYVSSETYRLNVKNGTETYLQVVSSWVPFNKNKMVGYLAASAYQSYAAIYGGGWITSFDTNAMVIMVFFRAELELLRIDCKDIFGTESAPVEHGIALKRLKECHRRHVELVKYARLFDSCLSPIMLLYMFVCSVMLCVTAYQITIETSPMQRFLTTEYLVFGIAQLFIYCWHSNDVLYASADLMLGPYESTWWSRGVQYRKSLYLLVAQFNKSIVFSAGPFTKLTVATFISILKGAYSYYTLLSQSQMK

>SexiOR11

MFHLDTYAGDAVVGISGPMDYKYMKVLRFVLRIISGWPGKALGEKTLKIEGMGHAYYNTILSLIYLALGIAYLKKNAHRFGFLELGHLYIVLLMNMLSTSRAFTLCLSEKYRTVAKIFIQKVHLFYFKDNSEYAMSIHVTVHRISYFAAVYVSIMLSIAACLFNLIPMYNNYAAGNFASFDNLQNTTYEHAISCLYPWNFETNFNGYLVATLSGWYGTMLCGSSVSMFDLFLCLMIFNLWGHFKILIYNLDHFPRPATEVVDAEGEERSGRIIGSEMYSESELEEVSVKLKECIQYHMLIVDFTNNMSDAFGMALFIYYSFHQITGCLLLLECSQMTAAALSRYLPLTIIMFGELVLLSIIFETIGTMSEKLKDAVYKMPWEYMDTKNRRTVLIFLIKVQEPIHVKAGGLVDVGVTTMASILKTSFSYFAFLRTF

>SexiOR12

MKATLRKFGLEYCELPEMLSNVSTMLRMLTLNVDSRNKTPISTIFYVLMAIISTNYIYVYVISGIWFVFWRCYKTGDLGAAMIVFSLMSTSEVALIKLFYMIFYEEKFKNLIDKYLACDSRTVKGSRFSKNLRKALRNVKMRGIICWLVLNMNAVLYVLRPMVTPGQHLTVDAFVILGLEPMFKSPNYELAVFTTMISVFFICFTVANVTCFLIMITGYTESQMLALAEEMIHIWDDANEYYQTMIEELSSYESVYLRNKKLESVKETEVLNEYVAEHLKDIIQKHSFNVTLLEEIEDVMRGPNAVGFLFLIVGLIAELLGGLNNTILQVPFTLSQLGTDCFLGQKIMDANIKFEEAVYACKWENFNKVNKKIVLVMLQNSQKTMTLTAGGMATLSFSYFMNIIRSTYSAYTTLRSTI

>SexiOR13

MGIMMSKKFLKMDLKLSTVKIFSDGSDLEGIENVEDIVYLRILKKIMWVIDGWPKEANKSQFFRYYICIL

DMVSLIPGTLYLKINTGKIPSFELGHTYITVFMNAIAALRTVLVLTKRYNEIIFYFLKEVHLFNFRRKSK

YAYETHILVHKISHFFTMYVFMLMCCGILLFNLTPIYNSYAAGMFKDERPPNATFDHAVYFALPFDTATN

FKGYVVVSLYNWYISITCSTYFCIIDLTIFIMVFHLWGHMRVLTYNLEHFPKPASVMAAADDPNAYKNCE

NKYNEEESVEVFLRLRDCIKIHSLVINFSSMMADSFGWTLLVYLFFHQVSGCLLLLECSQLDTAALARYG

PLTIIIFQQLIQLSIIFELLGSSNDRLIDAVYSVPWEYMDTANRKNVFFMLRQSHRSMNLKACSMVTVGV

QTMIAILKTSFSYFVMLRTVADEEE

>SexiOR14

MDEKPEFTTFHETYKLITFALSVGMIYPNPKTELWRLASIPILIASISPLATMIFIDMYKCWMANDIVNIIRHSTVVGPFLGGFFKMILMYHKRIQAKQILDEINRDYKNLNNYSEIYKEIGRASVKNCQIYSERGWAITVVTCVMTFPVMAISLNIYNFAFKSEPVKYMIHDLEKPFSDNPEARFESPYFEIIFVYMFYCSILYVVNFTGYDGFYGLAINHACLKMDLYCRALEEAFKADAEEVCGRVIGVIKEQCRMFQFVDLIQDTFNIWLGIIFLATMIQICTCLYHITEGYGFDLRYMIFVTGAVIHIYLPCRYAAKLKAMSLETANRFYSSGWEQVDDQRVRKMILFMVARAQVPNEIVALNMLAFDMELFVSILQTSYSMFTLLRS

>SexiOR15

MSESTIEQAKREIDESLVLSAFCMRRIGLSFQDPKSASAYLRQKLMFIASVCGICYHVFSEIAFIGLTLSNSPRVEDVVPLFHTFGYGALSIAKVFVLWYKKDVFRQLLNELAGIWPMPPLEEEALIIKNKSLAALRITHRWYFFMNVSGVWFYNLTPIAIYLFALIQGKNDKIGYVWHSWYPFDKHQTVAHVAVYLFEIFAGQTCVWIMVSTDLLFSAMASHISILLRLLKRRLESVGTADNEHYG

>SexiOR16

MNLKKFLFENEAVVGISSPSQYLYIKIVRFVLAIVGAWPKKEIGEPEPRYQTIMINTFYFGVVNAALFGSITYVYFHNSELSFLEVGHMYIVILMSAVDVTRVYTLTYSQKYRDLAKEFLTKLHLFYFKDRSPYAILTHKKVHLICHLFSLWLLSQMLTGLSLFNLIPMYTNYSSGRYASGGTQNATFEHSLYFPYPFNTSTDINGYIVACILHWLLSYFCATWFCMFDLFLSIMVFHLWGHFKILINSLDNFPKPSTQVSCTLEGGFIVNAEKFSKEELIKVSRQLKECIDYHREIIQFTNTMSDVFGPMLFCYYIFHQTSGCLLLLECSQMTAKALMRYVPLTIILTQQLIQLSVIFELVGSESEKLKHAVYGLPWECMDANNRKVVAFFLMNVQEPVHIKALGVANVGVTSMAAILKTSMSYFTFLRSM

>SexiOR17

MCLYLPFDLTIVILTSNVSALLRLLQMDLENAIKIHDEDQNLKKNINMTDPDSYENVRTLVIVHQKLLRISDQLSSVFGLVIFIHVAFAALEICFFGFLTLVYGGVADTIANLLTVFNAVFTIFLLSLSGQFLCDTSSQVADAAYQSYWFESDVKVKKLILFIIIRAQRPSYLSALGFSQLTLKSFSKIMSSAWTYLSLLIQVYEET

>SexiOR18

MFFSVSDSTVLDGVCVPRGDARSITNSDSIMTSVYLSDWLSASPRFRRHLLIAMLRWSKSITPRVSGIIPLSLTTYVSVLRSAYSLFAVLSTKH

>SexiOR19

MRDKIIEALNLIDCDEFVGDYEKKGGLYKTNMGFKLGWKSYLILSNIAYSSQVVAPVFLDILRGTKTELPICKYYFLSDEVRESHFLFWFLYQSCGMYGHMMYNVNIDSIIAGLLLIAIAQLKLLGNNLTNLKLSDEERKLSKDIQDKIQITRFNKMLRHYEVILNYCDTTQDILSVTLFFQFSVASIIICVVMCGLLLPSSTETKVFLVMYLFTMTLQIFVPGFLGTQLTHASEGLITAAYNSEWIPRNESYKSSLKLFRERISRPVVISGLKMFPLSLLSFTSIMKTAYSFFTLIRNVQEA

>SexiOR20

MVAKMDASDLNFEEMFKIATVSMRLNRSHPSIIRDRRWKLQFSIIMMITFFCFLFLSYSIVYHDIKSGKFAEASKNGTMVIVSLTITLKYVVLLHHQESIKELINIMERDYATAQEFCNEDKEIVVQYAKRGVTVRKFWLVFGFGTSAIFPIKAFVLMGYYYWKGEFVLVPLFDLTYPQPIEDYKNVTIVFWLLFLFTFSFDAYASSMYVGFDPMVPIFMLHTCGQLDLLSRHISTLFVNANHEEIERGLKKIIAKQQDLYKLVDRVKKNFSILYEYNMKATTFLLPLTTFQIVEDLRDKRINVEFISFFVGCILHFFMPCYYSDLLMEKSEKFRQAIYSCGWEKCPNRRIRQIVLFMITRARIPLGITTVFYEINLDTFAEMCRQSYGILNLMNAAWE

>SexiOR21

MLTFKEIIHEIRKFGLEYCDLPTMLENVSILLRVLTVNIDSKYKKGITILSYIVTAVTAACFYYVFLFSMTWFVFWRSRITGELVGAMVVLSLGISSEIGPFKLFYMCYYMDKTQKIADSFLECDANTIKGTRFHTNLMKRLRSVKKRAMLYWVVVAGNGVLYVMKPVAMRGRNLPENYFLIYGLEPMFETPNYQIAYFMMIASVFFVCYVPASVTAFLIVITGYAEAQMLALSEEMLQLWSDATNHAKAQTEGMSEELDVYDPKVKMIINQFVERRLREIIGRHANVINLLNQVEIVFRQAIAIGFVLLVLGLLSELLGKLENTFLQLPFALMQVSMDCFAGQRVMDASAMFEASVYDCKWENFDKSNMKLVLVMLQNAQKTMTLSAGGVRTLSFSALMSVFRGIYSAYTALRSTMK

>SexiOR22

MVSSEDLFLNRAKFVMKYLGVWVLSENASCFLKAYRAFMITLQYLFLIFQMIYIIQVWGDLDAVSQASYLLFTQACLCLKVTVFQINMPMLKDLLRLMDAKIFKPENDTHEKLLELQAARIKRLLLAFMISSQITCGMWALKPLFDDADRKFPFDMWMPVSPEDAVQYYIGYAFQLGTICISAYMYFGVDSVTFSSVIFGCAQIDIIKDKLMSITSIKGNRGSKEVDEALAKNYNKLVDCIKHHQAIVKFTELVENAYHSYLLFQLIGSVGIICMSALRILVVDWRSMQFFSILTYLSVMISQLFVCCWCGHELTATVRK

>SexiOR23

MMETLRRFGPQYCDVETMLWNVSVMLRGLTLNIDRRNKKPIPIVVYIVSISICLGYFYVYLVSMSWFVFHRCQETGDLLAAIIVFSLGVSSEIGTVKLIFMFLHIGKVRRIVSECLECDSLVVAGSRFSTNLLRTLTVVKKRALMFWVVIIGNGVVYVAKPIILPGRHFTEDKFILYGLEPMDQNPNYQIATILCMAGVIFTCYLPANITAFLIVVTGYIEAQMLSLTEELLHLWEDAESHYYVTHQTNAVLEENIENTISRNKVINDYIESHLKDIIKTHGRNINLLHQVESVYSGAIALEFVILGVGLIAELLGGLENTYLEIPFALAQVGMDCFTGQRVMDASLKFERAVYDCKWENYNLSNMKIVLMMLQNSQKTMKLSAGGIIMLSFSCLMQVFRSIYSAYTTLRSTMK

>SexiOR24

MDANTKTTLNSVKPKHYIHYIEKPLKLVACWDFFPNSATENRKIFNDIYLGIVLFVLTHIPMVLTVHLYTEWQDIMSSLGTIADALPLLVSLVIVAYYAIYRRDLYELLNYLDTNFKYHSARGLTNMTMEQSCMTARRFGRIYTACTMFSVTMYATLPVIVHLWTKEPIQSWIYMDVTQPPFFEFVFLLSCLAQMYVGLAMGQFGVFFASNSILICGQLDLLCCSLRNARYTALLQRGVKHAALVATHSDIQRDEDHNYIYNIAEIKESAYHYDDRVTHNYVDVKTHFDIYSPDYDDATMEALRDCASLCQVVNKYKEMFESFVSPLLALRVVQVTLYLCTLLYAATLKFDMITVEYLAAVALDIFVYCYYGNQIILQVCL

>SexiOR25

LDVTVTYFIIPGLSPKYETPFYEITFVLTCVATAFSAINQTGYIVLFVTLICHELGHFYAITEALDEIHTILIKNERSRNNVGEGTGRESVDDLLMFCVKHHQFLMHYHGKIRELYKVIFGAHFLSMTVVLVTTLQTMNAWDVRNTILTGVTGIMPLFLYCFGGEMLISAGVQMSTSVYGCGWELMEAKQARVILLMLCLSQRPLYLTAADIFVMNRETFGDVAQVVYKIYAVFN

>SexiOR26

MPKQLLLDKSLDKLGVLFRYSGINLEKDIVSPMDTIKHRWLYALNNFCVVTAEMVGIYYIIDGIIKGKSFIEVTSVAPCLTFSLLALIKCMYHHMYEEQIKELINLLRDLERKENDREECADKQEIIDEEAGFLNKVINVLYVLNCCMIVVFDMTPIVLIAVKYYKTKQFEMLLPYLDVFTIIPYKLAYWPVAYFYQIWSEVMVLLSMAAADYLFFTYCTYIRIQFRLLQYEFERIIPERSISKGERFDEAEFRDKFRELVQWHQDLIRLLTYGVHMVIPRPQKKPAIP

>SexiOR27

RASPSRSPSTWTRTGSSSSSRGWTVSARHRAEEARGPSGTPPPPFVAEPLLNQSAEPRGALLRGTARRAARLLRVYSGCAVATCVLWIVFPVLYRIRGVAFEFPFWAGIPYNNNVVFSAVLLYSFYTTNLVAIGNTTMDAFMATILDQCKTQLRILRMNFETLPERARALQLAGGAGAVFEASLHRLFVDCLLHYNIITDTCTVLHDVFAIPLLIQFGVGGWILCMAAYKIVSVGI

>SexiOR28

MALQVCQSSGSAVSLLLQIALSDQLTFIASMKIIFFVVALFFLLGLFLCNAGEITYQASQVSDAIFYCGWHTCPMRPKSAPRLNIRQLVVLAIMQAQRPLVMKAFKMLELTYGTFLLVVRSTYSVFALFYAQTS

>SexiOR29

MGLLLPKSFLGKTLYIFLHGLVTLFVVTQYMELYVIRSDLDLVLTNMKISMLSVVCIVKVDTFVLWEKHWREVLEYITEADNFERQSKDPTRSQIVEAYTRYCRRLTYFYWALVSTTFLTTTCSPLMRYLSSSTFRENMRNGTEPFPHIFSSWMPFDKYHSPGCWITVLWHTVLCAYGAAIMAAFDTSVVVTMVFLGGKLDLLRERCKHMFGSYGTAISDPQQYEQEVRQLHSIHMMLIKYSRLFNSLLSPIMFFYMVMCSLMLCASAYQLTSAQNAAQKLLMAEYLIFGIAQLFVFCWHSNDVLIKNENMSFGPFESNWYTASNRQRNDVLLLLGQLRIQNIFTAGPFANLTLPTFINILKGAYSYYTLLRK

>SexiOR30

MFNNTLIFLLIIFELCSYFTQTGLTEKQQSNRLIYAISHPMLFMFRVMMTSIKERVRLVMYSLTVGLKRVHNDPEVEKQMIACTVMYLSALLLSCLMSMIMYAAEGFWEVVRNGNTFTTIITAYPAVEDDSDMANLVRAICFIIWWMFLTRIFAVYILVISLTTCLSYQYKNLQSYFLSLNDIFERNDLSQVEKENQYEAGFKVGIKLHADTLRCTQLTQSVCRGVFSGQIIFNILLLVVLMAQMANSERTLVNLCSAGFTATAVLISTGFYMWNAGDVTVEASHLGTAIYFSGWYHCQGLSSVRIRKLVVLTMSQAQAPVVLKGLGYIDLSYQSYIKIVKSSYSVFSVIF

>SexiOR31

MGLHFLYWLPFDPYQPVYYEITLVLQTWHALVVIWFNVAWDMLFCLFLCHITTQFDLLARRVRRLFYVQVDNQLVSSYPMASVSREFIRSEGERVNSYSNQYWEARYQKEITEIVLRHHSLIRLTGDVENMFSLALLINFMNSSIIICFCGFCCVLIEKWNEVAYKSFLVTALSQTWLLCWYGQKLIDSSQRLADALYGCGWYNSSKRARSAVLIMLHRAQKGIYVTTHGFSVISLASYSTIIKTAWSYFTLLLNFFKEKSVY

>SexiOR32

MVLKFLTLLEDPNDPLLGPTISMLRFGGLWQKNRLKNFLHNLVHFVAFIFVLSQYVELWVIRNDLEMAMRNLSLTMLSTVCVFKACNLVFWQNTWKELFDYISELERSQLVKKDDTINKIIFKYIKYARRVTYLYWGLVTATVLTVSLAPLLTYWS

>SexiOR33

MYETPNYEIAWFMMTASIAIICYISSNVTAYFIVIIGYAESQMLSLSDEVTHVWEDAEKHFETLNLGLEFQDFDNKKIIKNEHVFKNLKYIIKRHATIKTLINQVEDVCSGPTAVGLTFLLLGLISELLGGLENTFLQLPFAFMQVGTDCFIGQRIMDAGEVFERAVYDCHWENFDPRNRKMVLLMLQISQKTSSISAGGMTKMSFRCLMGAMRVTYSAYTAFRSIM-NLNVRIEVKECS

>SexiOR34

KHQKLLELSRQLDNVFNGIVFIDLLFVGITTCAFSFMGQFARGPGYMLISYIGIASSMFTILYLCYYGELLTSASSSIGDTAYENLWYQGSRRYKMAIYFIIKISQKPCCLTSIRYAEVSMKMFTKVVSTTWSYFSLMNSVYSEES

>SexiOR35

MPNRFISTRSCNRILYFVEIFYVIFATTILGSLFTQKNLSEKQKMDQMMFSVTLPGNIMFHYILLFRRHAIRNLLYHLAVVLKVHYNDPDLEREMIKKIKVFSVSLCGLVGTVVVSYGLSAFYRVITAGETFVTITSAWPNVHDKSLAAGVVRVFVYFWWYPFAARIMVTFLMLVTMLVSICYQFKNLQSYFYSLDEIFCDGTLSQEEKEKKYEDAFKLGIQMHSLTLWCKSQHQHVSKELFATEILLFFGMLLSQLTALLANDRNMTQLWTMFITSVTTCLALGFFMWNGGDITLEASKLSEAMYCSGWQNCCGQSSVRIRKLVVNALRQAQNPVIYKTLGIVEFSYESYVRLVKMPYTAVSVFY

>SexiOR36

MKSKLAFLSPVVPYGVFESWEDLNPRLYHAVHIYWLKFYGMWFNDFSPKSIKFWVQMMYTLIVLWLVCFFPGTGEVVYLLRRRENIGDVAEGLYLFLSEMYTYFKVAVFWLNRNKVIELLKYLHCEEFKPKEPEHRDMIIKSIKSARFVMTYYSTMCVGAVSVGIIMPLTENFDILPTNVEYPFFNVYQTPAYEAVYIHHIYYKPATCIIDGVMDTILAAFVASAIGQIEILAFNLRNFEVLADRQRKRDVAENKYIEEYKPQYYVRSVLKDCIVHHNCIIRYVSMIESAFSLASALQFMLSVMVLCLIGIQFLSIENPSSHPMQIAWMGIYLTCMLIEVFILCWFGDELIWKSMDLAKAAFEGPWMNSDRKSNMFIIIFLERCKRPMRLTAGKIFTLSLDTYTVLINWSYKAFAVMRNMKK

>SexiOR37

MYGFTAFIFIISPFVEYILRKIRGMECLAYPHILPGWAPLDDLSFVGYMVTVVGEFLAAVYCVCVHIAFDLTAVGIMIFVCGQYSLLRDYSSRIGGKGDHCNLSKKRDLRAHYRIVRCHQTNILLVNTINDLDVLLKNIIGVYFFVATLTLCSVAVRLKYKFSGRDEYNAIGLPNPIHVRYINTALYILHIRGRSIEREFDGDGTGTVCSCLMVFKSSNQKRPGDPRLRYDEATAPSCWAI

>SexiOR38

DNEHYGEILANIKLHQRLITYCNDLENAFSLSNFVNIMLSSVNICCVVFVIVLLEPFMAISNKLFLGSALIQIGMLCWYADDIFHANADVAAAAYNSGWYRTNPRCRRALLFLIQRAQKPIAFTAMGFTNITLVTYSAILTRSYSYFALLYTMYNEN

>SexiOR39

MEYEYKSYDELKQDFLGEMDFISNLGIKMFIYPFIGRSKMIMYCFYITYGLLFLTSAQLIVTLCIICIKNFDWFEIINVAPNIGVCLMILIKYKKINDNKELYNQIFKHFRFDLWETVFDTEKHKKILNRYTKTTRLILRFEFYYTIGLAIIVDLFPRIIMIYQNDIVGMEKQYLYPFDGWYPFDKIEWYNAAYIWESFMTTVVIFIYVFVNMLHISFTRYICMELKILGSTMEDLITDDDVVKIKKGREVAKIHKKISNKLKFIISKHQYLARITSDLDKVLGDGMFLTYMFGSVFICLTAFTATVTTFSLDNILSIIVLYLLILLFVSFPGCR

>SexiOR40

MDKFLGRSSSTESLATKRPAEDEIWRKPKKVNINARRPLQRPVYDVDSYDATFAITKAILRLVGLRITRDDSTFARLLWNIFYWFEFGNLFVVTWLELINMAKTARGGSFQDAVEIFRMMPCVGYLLLAMAKSYKMVYQRPVYENLVNELRSMWPRGEVSDEEHQIISSALRHLNYVIQGYYWCNNALLVIFLSPPFVEILKIAMGHDTPLILPFFYWFPFDPFQRGYYEVILAAQTWHGLITIWFMLCGDLLFCIFLSHITTQFDLLAVRIRRLVYVPVDKQLVHTYPLGEYCQGYAQKNKDIIETFTDKDWETRHQRDLSEIIERHRALIRLSGDVEHLFSFALLVNFFNSSIIICFCGFCCVIVEKWNEMVYKSFMTTALSQTWLLCWYGQRLLESSEGLSDALYESGWYRASQKIKSSVLIMLHRAQKDVHVTTYGFSIISLASYTTVSMFAISLNRYM

>SexiOR41

MLNYMVTMLSQLFLYCWCGHELTIRSENLREWLYQCPWYEQDTKFKRSLFIAMERMKKPIIFKAGHYISLSRPTFVAILRCSYSYFAVLNRVNTE

>SexiOR42

INVTDGEVSSSLTHANKKDVMKLNQCIIHFIKICRFCKLIEEVFSATLFVQYSAASCIICVCLCRFTMPAEMGYIIFLGSYMALMILQMMAPCWLGSRLMEKSQLLTFS

>SexiOR43

MMYNQIDCFKIHLTILRVLGVWSEENPSIYYIYFSRIFVFIFTVLFVVIYTMNFYFLPQQLEIFAEELIFYFTNVGALSKALAFIFLRDKVKKMLDMLESEMFQTDNPEEVKLIEKAKEKSLFYWKITFGLSVSANTVNVFLPFVLHLIFPIKLEFPVCRYSFIPEQYEAIFLYPAYLYQSIGITSHMLYNVNIDTFLLGVMFLAMAQLDILDRKLRKVTDVCLNVDAPRGSIDKMIDDQNAVLEINKCIKHYDAVCE

>SexiOR44

MRVLSYVWKRISQSKALELAGPLETAFFASVYRLSFVVGLSTSDDYLLYTAYSSFIRIISALVVWFEIWSVLGNNDVSLDQIISSVNVIFIHLVTFWKLVTMVKNKRVFKKLAKALESSSFDMSTQRRKGIVNYWVLINNKYLKVVLTLGCLTLIVWELYPMIDELKFNLMVDVKLPFEYQSLLTYMATYTAVAIMFAYASLMVIISEVIVQAHLIRLICQFDVLADCFENIFEECAEEFPDLNKHELVKDASFVDKYVKRLGDLVTQHREILDQTNDLRTILSAPLLGQLACSGLLICFVGYQATATIAENLGKFVMSLLYLGYNMFTWYLMCRWCEEITIKSQRIGQAAYFSGWESGISLAPGARASIILVIARANKPLVFVAGGMYSLSLSSYTSLVKASYSALNILLTMSHE

>SexiOR45

NNEPGNFEDLKTVIVYHQQLISISKQLDDVFDKVIFINLSSASISICFFGFCAKVAHRAIDMVNNFVAVVTLTLPLFNLCDYGERLREASAGMADSVYHNLWYRGDIQYQKLLWFIMRRSQKPCCMTSLKYSPIGLNTFAAVLSTTWSYFSLASSLYESET

>SexiOR46

MDDKFEKDLKLFLIPMRLTSTSPEIPLTIKRFIRHLFGYIIFTAVAIVITYNTYISITNRIFFEACRNLTLSLTYFGCCLNTMLSFWNRRSLKTLLETIRNDYKMAAQLPPQEQLIFEDYSRKKSLICKIWFHLFVVSLCLFVLKAFTLMVYYYFIGEFRLVHLYELTYPDYIEKRKGDLVMYLFIFLMVFLYGVISGLGFLSFVPYGTVCMLHACGHLEIAKNRIDSLFTGDSRYVNEKLKNIAQLLQYTYHFIEYSNGCLRFFYDAILKLSAIAIPVTFYALLEGLRHGVFSMEFTMFILNAIMLTSIPCYLSDKLLEKGEEVRLALYSCGWECEYNRRMRVTILLLQTRCSRPIAVQTMFTTLCLYAVTDMFQQAYTILNLMNAVWN

>SexiOR47

ILSGIGTYQNKHDLVIFFSNLDKSLVIYKFFFKAIIFLIKRDELRDLIDEMEASGDEVTKERKKLMANYVMVITGMTAAVVFTFSVLALIEGTMSVEAWMPFDPRKNSMNLVLSLQIMAFCAFPGLCRAFAMQGLVCSLIMYLCDQLIHLQKELRSLDYVKDTEMVTRMKFKMIIKKHIRLMSYSMRMESIFNEYFLVQNLAVTVELCLNAVMVTVVGAQQITLLFTFLAYLIIALINAYIYCYLGNELIIQSEGIAQAAYESTWTSWPVDLQKDLLIVINASQRPLKLSAGGIALMSIQTFSQALYNGYSIFAVLNDAVN

>SexiOR48

MEEEPLLINKTIKKIEIWFRATGTNVKSTPKTRMDTIKSRAIYIINLLWLNMDLSGAVVWFISGIANKKSFTELTYVSPCITLSFFHFKCFFLFLNEDSVDKLLEKLRELEMNERARPKHKDKEAIMIHEYKFLTNLISFLNLFFCGLIVAFALGPVAITIFIYVTTNEVDLQLPFLIIYPFDAFTMKYWPWVYLHQIWSEVVVIVGLGAADYLFYTFCSNISVQFQLLKYNIEHLIPDDETCGRLPNIEEIRTEVVDLIKWHQDLISSVKLLETIYTRSTLLNFVSSSALICLTGFNVLAGSDFVYVMTFVSFLFLSALQIFFLCFFGDLLLNSSTKVSDAVYNCRWYLAGTSLGKDLLLVQIRSQTPCKLTAWDFSEVNLKSFMKILSTAWSYFALLQTMYGSSP

>SexiOR49

MLLFHQDTVVELIKNVNSDYKELPNLSDIEKDLMNKYVKQGVRVCRQWFVLTVAGTLSFILKSGGLMLYYYFINDFRYVSLYDVKFPACIEERRNDHFFVFLATYFFLMFFACYSALNYVAYVPLGPIFMLHACGQIELVRNRVEVLFSGSDVEEIRMKLKHIIKKLQYIYSTVDDMKRVFKFGYEITLKGTAVLLPITFYAVLETAKNGEVSLEFITFIIGGIMISAAPCYYSDLLMEKGEALRLSLYTCGWEMQYDRRTRTTLQLMLQKALRPVAIQTIFRTLCLDALTDLYQQSYAIFNLMNAAWN

>SexiOR50

MFEQKCCGPVVCLTAYCIAEAFDEGEFQAILLLLCIGTTVLHFVPCYFCTFLASKVSSVCDACWNIPFWNAGPVIRPYMVLIMQRSLRHLPLQAAGFEDISIETFSKKMTNAYSLFNMLRQANI

>SexiOR51

LGTAIYQMYFAEDFVDSINSFFNISVFILIGNDSWWLISKRNDFNDLLKMVITNDNLIIEAGRFLDVHQKLLKSIKIIVIMCYMFHFVNDVMIFIPSRTIGMDDFSTVSCVGMEPLSNSPNRQACMAVLALQELTAIVAVCSYDVALLFLFSHTTVVFQILYEDMTDFANISKSHETYYVVEDRLKNIVFRHVL

>SexiOR52

MGVIVKNATQSVSISLTALQIVGFWAPENITETQRSMYKWFGVFSFMFMLGTYIFIQVVDLFMIWGDIPLMTGTAFLLFTNMAQAAKIVNITVRKQRIQNIVNDSDKVLSEVQTLAEKEIVKSCNREMIVLQVLYFSLTLVTGLGWATSAEKHQLPLRAWYPYDTTKSPAYELTYVHQVGALLIAAFLNVGKDTLVAALTAQCRCRLRLLGLSLRSLDKGLDIEDKYMLTAEQEKIVSCRLRSCVLQHQETLKAAKELQECFSEPTFAQLTVSLIIICVTAFQLSMAQPDNMVRLLSMGTYLLNMTFQVFIYCYQGNQLSEESFEIAGAAYECHWYKFSLRLRRALLIIMVRTRRALRLTAGGFTTLSLTSFMAIIKASYSLFTLLQQVNEE

>SexiOR53

MKYHQIDCFNINMKFFKFLAICPDNNPCHYYKYYSKVFIMVFVIIFYLLFTINFYFLPRQLDIFIEEMIFYFTDLAVTSKVLTFVFMHDKIIEILDILECEVFQPDDDEGIIILKKAKDFNKSYWKIIMSVSYLSSLVHIMSPLISHLILGVELLLPVCRYAFIFDQYKLMFIYPIYLYQSASIHFHMLYNVNVDSFFLGLMVLAIAQLDILDVNLRKVTAENKIEDTDGEMSRSPIHRAKEDVMKMNQCIIHFIQVCRFCELVKDVFSATLFVQFGAASCIICVCLLRFTMPAPMGYNIFLATYMTVMILQIMAPCWLGTRIMVKSQLLTFSVYNCDWTSRSRQFKSNMRFFVDRANKPLSITGGKMFILSLDTFTSIINSAYSFFTLLQHFQKE

>SexiOR54

MMSVFGENMIRESTKIGDAAFLCKWYKMDEKSKKIILTIMIRSKKPQQLTAYKFSTISYASFTKIISTSWSYFTILRTVYSPPEVTRTE

>SexiOR55

MNFFKNPNIPTEQYKETNTTRLIDKYSKFWFLCCCPDFWVKKVNYPVPFTRVYRPSMLINHIVMVIFCSSCFLSLWTQDGLSQSQKSDRLAYAASTPIITVLYHFVILYYTNDVRQVFYKLAVVLKVDHNDEKAEEEMIKQSKFHNGVFFSSCVCNMILVGLYNFIRAVTSDATFITCISAWPDIEDRSTLAGLTRVVVYFLWFSHVTRNMGVFLIIHTVLLLLSQQYKNLQSYFEDLNKIFLEKNLTQEEKELKFEVRFKRGIEQHALTLWCVDETQRIFKITFSSHALLWCGLLISILPDVLNNDTHSPTMLVSNAPRVCAALVGLGYFMWPAGDITVEASNVPHAMYGSGWQCCHDRSSRIRKLVVLAMMQAQVGNF

>SexiOR56

MLFVGFTIQSVFATLLFAGSFIYVGQLSQLIYLVQVFPDKVETFKTINCISASSVPLSKFFFMWCARYFL

>SexiOR57

MKILSEETIEKYSKTIGDRNEVDKLVILPLCVQDILGHNVLNPKWKWTTRIPQQLFLVFLIVYVILGTNDYLKDATDINDIGEAYYTFIIILFFPIKYMLFIQNRFTLRQLYAMAKTTLLDMIKSDADEKLEELFAKIKLAVKILFGTVFWSISVYFMVAMWNYVQGTRVTLSKSTTTLMPMTSPYYELGLAIHTVFLFEMAFTYCVIDFWFVLLMFSFCTAIDSTINRLKIEGKKSDETDEQYMDRLNDALRIFYKNHSKIMEFLYILSETYKWPSIIPILGILLAFCLILLCMSVEVHWMFLSNLIPTTTELFAYNWFGDQVKDKGTALILALSEFDWPNMRNKDKKNYLIMVSYMNKEFRIKTALGYDLSLVTMSSVFKASYQTFAVLKTVDH

>SexiOR58

MSDKCSIQNVLKFLEDPKYPSVGPHLKLLGFTGLWHPNRQTVIGRFKQMLFYVTITFFFSQYVKCFIKFNTDSLKLILQYAPFHMGIVKSCFFQKDYKKWQVLIDYMSSVERDQLAKNDKDHEKIIHEYIKRNRQVSYFFWALAFFSNFSIFTEPYQKNQINVNGTSVYLKIFDGYTPFDNEPPGYYFSMLIQTVLGHIVSAYVVGWDTLVVSIMIFFAGQLKITCMYCRLAIDLLNSSKSHDNIAQCHRFHTTLVEYTHLFNALISPVMFVYLVVIAINLGVCIIQIVEIHDDIATLVSSILFVVACLIQLLLFYWFANEVTIESTFVSYSTFESDWPQANNKLQKEIALLGLTTKKILVFRAGPFNQMSLTTFIAILRASYSFYTLLNSTN

>SexiOR59

MILMTFIDISRILMLTFSKEYRKVCKEFLTKVHLFYFKDNSEYAMKTHKRVHLMSHLFTLCLLSQMIFGLSCFNLIPMYNNYVAGRYKSGGTLNSTFEHSLYYKYPFDTLTDIKGYILANAINWTLSYLCATWFCMFDLFLSLMVFNIWGHLKMLIHTLNNFPKPRSDRSYLFQESSIETSKKYSEEELLDVFKKLKQCVDSHRLIVKFTNKVSDVFGPMLFCYYLYHQTSGCLLLLECSQMTAPALMRYLPLTLMSTQQLIQLSVIFELVGSESEKILSAVYSIPWECMDTSNRKFVSFFLMNVREPIHVKALGIANVGVMTMAAILRTSLSYFTFLRSI

>SexiOR60

MLQDIFGTAILIQFGIGGWILCMAAYKLVTLNVLSVEFASMTLFISCILTELFLYCYYGNEVTDESERVSESLYSMEWGRAGLAFRRSLVLVMERAKRPLRPAAGRVIPLSLDTFVKIIKSSYTFYAVLRQTK

>SexiOR61

MRLQILKKFLLKESFDFDRPDINLYNFHPQLRIFLAVKGVFFTNRSSRLRLIWPIICIQLSIIGMTFEVIFIWRGVTIKDYSFATESFCYWLLLGCIPIVLASIGVHTNKIYDIVVKMNEEFIYVCSLGPMYRKPFLEGQLLIWQLCYAWFGFVSFVGGLYVVFPLVGLIYQSLFATLDENTTRPLQFPMWLPHDDPYRTPNYEIFLLIESTLCFCFVQTFCVYVYTLFHILLHYYMIMDMIIIDFSVIFDGLEQSVALLPRSDTRRMETQRILNARIKKIVKWHLSVFRAVKTVSSIYGPPLVYQVSFSSLAICLIAYQIAELWIPCYLGTMIRNKAFEVGDAGWTCGWHETPLGLMIRTDIVIIILRAQQPVTIKYTGLPQIQLETFSSCMSSSYSYFNMLRQYSK

>SexiOR62

MTLAVVLILFFVKTYYAIYATQKFAPLLDKISEDLLEANNLGERQQKLYDEHIKIAKVGEISWLLVPVLMSALFPIYAGTLMSIESIQTDDYQRRMVHDMELLYVEDIQSETPFFQCMFAYNCVQCVVLVPNYCAFDGSFCIATTHLQLKLKLMALKVSDAFKYSKNKYELRMKMNEAIRDHQDALDFYVQTQNVFGPWLFAVFLLTSFLISFNLYQIYLLQRIDPKYTSFGVVGVLHIYLPCQYASSLTKVSEEIPNDLYVVDWEVWADPEVTKQLIFMITKAQKEMVVTGMGLVVFNMELFKSIMQTSYSFFTLITA

>SexiOR63

MVPCMTFIIQTLFHLSGLEPMFKSPNYELAVFTTMISVFFICFTVANVTCFLIMITGYTESQMLALAEEMIHIWDDANEYYQTMIEELSSYESVYLRNKKLESVKETEVLNEYVAEHLKDIIQKHSFNVTLLEEIEDVMRGPNAVGFLFLIVGLIAELLGGLNNTILQVPFTLSQLGTDCFLGQKIMDANIKFEEAVYACKWENFNKVNKKIVLVMLQNSQKTMTLTAGGMATLSFSYFMNIIRSTYSAYTTLRSTI

**IR**

>SexiIR1

MFPFIFLIWLQMVNNYKRKSNRNSWDEASMKNAIEKVKRDAKPGLYLFGEIINSILYTYGMLLVVSLPRLPTGWSIRLLTGWYWLYCILLVVSYRASMTAILANPAPRVTIDTLRELVDSKVTCGGWGTQSKKFFQESLDDNTQKIGEKFETIDDPLKAANKVAEGVYAYYANSDFLKYISVVRKTALKGSNDNSTNTTEVTPKKDTQRNLHIMSDCAVNIPISIGFHKNSPLKPLADVYMWRVVEVGLVEKWLNDVMYPIHSLETNEDEVKALMNLKKLYGAFIALAIGYTLSALCLTGELIHWHFIVKRDPNFDKYALHLYYRKKNY

>SexiIR2

MAGIELIISSICNATFCEIPYNETYQAPDSLVEKDINFMSLMKEVNGKHIKVTTYNNTPLSSTEFENGTVVGYGVAFTIMNILRKKFNFTYEIILPTKNYELGSKISDDSIIGLLNTSKVDMAVAFLPTLLPYREKVSFSIDLDEGVWVMMLKRPKESAAGSGLLAPFNDLVWYLVLAAVLTFGPCITFFTRVRSKLITDDEGVLPLKPSFWFVYSAFLKQGTNLSPEANTTRVLFVTWWLFMILLSAFYTANLTAFLTLSKFTLAIETPRDLYQKNTRWVASAGSSVEHVVKTEGEDLYFLNAMISSGKARFLSVSGDKEFLEYVKKEAVLVKEQTVVDHLMYNDYISKKDVDESDKCTYVVAPSAFMKKQRAFAYPVGSKLKSLFDPVLTQIFQAGILDFLKRSDLPSTKICPLDLQSKDRKLRNSDLIMTYLVMVAGSATAVAVFGAEIFVKRYLSGKLNKTKKSKRKKSKAGKSSKTHDDSRPPPYDSLFGKNPKFNLETTRMKMINGREYYVFETSNGDKKLIPARAPSSFLYRSDK

>SexiIR3

MRPEPSFYIIVGENGFVMDTYRRAVKEKLVKRNYRWNLIVTDYSSVEVSQLVLPTVILQSDPVECCRLLRKDDCSCPSDFQRKQQIINSLILYLSETYSKLEGDLTLTTSSIQCEEPQSSMNTTRDRLFKQFAEDSEISNETVFFWDGDRSGLFLRSRFILSTYKPEDGQQVIATWSADEDYKLLPGIKLEPLRMFFRIGTAPAVPWTLVKVDPTSGEQMYNEDGQPLYEGYCIDLIARLSETMSFDYEIVSPKTGDFGKKLPNGTWDGVVGDLMRGETDIAISALTMTAEREEVIDFVAPYFEQTGILIVIRKPIRKTSLFKFMTVLRTEVWLSIVAALVLTGFMIWLLDKYSPYSARNNPDAYPYPCREFTLKESFWFALTSFTPQGGGEAPKALSGRTLVAAYWLFVVLMLATFTANLAAFLTVERMQTPVSSLEQLARQSRINYTVVEGSSVHQYFVNMKFAEDTLYRVWKEITLNATSDQAQYRVWDYPIREQYGHILLAINASQPVPDAKTGFQQVNEHTDADFAFIHDSAEIKYEVTRNCNLTEVGEVFAEQPYAIAVQQGSRLQEDLSRALLELQKERFLEQLASKYWNESARQACPDADESEGITLESLGGVFIATLFGLGLAMLTLAWEVFYYKRKEKNKVQTFDTKPEKLAFESKTSLESKVAQSVAKIRKRGKIGKKSTVAKNVTFGDSFKPVSEKGVSYISVFPKDYRP

>SexiIR4

MQLWILGFVVLFTPVSGEDFPSLITANASIAVVLDRQYLGEKYQTILDELKDYIKELARVELKHGGVIVYYYSWTAISLKKGFLAVFSIASCEDTWELFSRTEEEELLLFALTEVDCPRLPSHSAITATFTDPGEELPQLLLDLRTSNAFQWKSAIILHDDTLSRDMVSRVVQSLTSQIDDESASPVSVTVYKMKHEINEYLRRKEMYRVLSKLPVQYIGENFIAVVTSDVMTTMAETARNLLMSHTMAQWLYVISDTNAQNGNLSSLINDLYEGENVAYIYNMTDNNPDCKNGIMCYCQELMDAFISALEAAIQDEFDVAAQVSDEEWEAIRPNKLQRRDKLLKHMQQHIAAKSRCGNCSTWRALAADTWGATYRSFTETTDIVNDNNMNTTNRVIDKIDLLNVGIWRPIDGVRFEDVLFPHIHHGFRGKELPIITYHNPPWTILQRNESGAIVKYGGLIFDIIHQLAINKNFTVKVILASVLKKELSNDTTTDMMHSMEAKLTISAIAKGQGALAAASFTVLADPVPGVNYTIPVSIQSYAFLIARPRELSRALLFLLPFTTDTWLCLGLAVILMGPTLYIIHRMSPYYEAMEITRQGGLATIHNCLWYIYGALLQQGGMYLPRADSGRLVVGTWWLVVLVVVTTYSGNLVAFLTFPKQEVPVTTISDLLANRALYTWSINKGSYLEMELKNSDEPKYLALLKGAELVSPTDGKSGTMSSGSSLLQRVRFHRHVIIDWKLRLSYMMRADRLEDDNCDFALSTEEFLDEKVAMIVPAGSPYLPVINKELDRMHKAGLIMRWLEAYLPKKDRCWKSSSMMQEVNNHTVNLNDMQGSFFVLFMGFFSASTVLLLEFLYNRRKRRSEQIVIKPYVE

>SexiIR5

MLCQAVLTSILFHVTLGLSQHAVQFGVEYFQYRGLKFICVMTCDRYFSWTVQYSKASQNHSMAISGVSIDSSITDYNRVENCLRRKLHPVGVIVDAGCGGTEDVMDFVSQNMLLDSNHKWLLIDDDEAYKVYNETEVDMIMADSGNGSLIEMLQRLNISVDADIVMGFREDTRYILQEVFNYGKVQGGNLIIHEIGSWDPDNGFNLSIDLNGYKYYRRWDFQNISMKFILVVQPVPDHFDPESLLGLEAVPGVAVMTQTSATILYAVAKMHNISYVPTITDRWIGTYEKNATRVVSNSLYFREQDLSPVIRFQLVQDRTDVLLPPLTSIETRYFYRIPTIGPGKFENQFLRPLSPTAWWSVIGVSALCSALLLVSALFEHRPSSVQYALFSVIASICQQFFQDIDDGGTQRISTARKVTILVTGLSCVLIYNYYTSSVVSWLLNGPPPSINSLKELLESPLELIYEDIGYTRSWLQNPSYYYNGKNAAIEDELRKKKVFNKKKGSPLLVPLVQGIKMVQDGGYAYHTEVNSANALISRTFTQSDLCELGSLKSMEKTVLYPCLQKHSPYKEFMTWSLMRLSEQGIVSCIQIRRLSFEVKCEGSSPRALALGGAAPAFILLAGGYILATIIMLIERMIFKINHANKIPKATAAA

>SexiIR6

MPVGLLIDGRCDQTQALLLQASRNKLFDAVHPWLVLTDIEDDNCTEYVMQTFRLLNLSVNADVAVVTNRGDSFTLIDVYNFGRIQGNDLETALLGNWQPDRGLDIVLKGYKYYNRWDFHNLTLRAITVIVDQPKMFYPEMLSEMAYTAGVAAMTKITSQMLNTIKERHNFRFNYSIAGRWIGSPERNSTMAVTNTLFWEEQDLSSTCARIFPKWLNWVDIYHPPTTNLQTKFYYLIPEKGVGQYENRFLTPLSHGVWCCAFFAGIACTLVLAGAARMEDRPKPELYAFFSVFAAVCQQGYEDGVQLLEQTISSQGRRLTLLVIGLTSMLLYNYYTSSVVSWLLNAAAPSIANLDGLINSDFELVFEDIGYTRGWLDNPGFFYYSGFKNVKEDELRDKKVTKAKRTVSVLQNVNKGVELLRTGKYAFHTEPYTASQVISKTYEDKELCNLGALQMMLPAHVYIMAQKRSPYKEFFDWSLLRLLERGHVKAIRARFAGTMPACSGAQPRALALGQAAPAFLMLAAFAMLSCFILVLELLWK

>SexiIR7

MGLVFHFFYYREHSNTTKWPTNSLLMTFSMLLGWGATFEPKSPTFRILIFAWLCFSINMGISYESFLRSFLMHPRFEKQISSEADLIQSGIPLGGREIYRSYFETNNASSFYLYRKYNSTTFSEGVRRAALQRNFAVVSSRRQAVYQDQKLGKGAPLIYCFPESNNMYKYGVAILTRRWFPMLERFNNIIRSVTENGLIDKWMNELLIHTVSSEEASTIVPLSIQNLLGAFMFIGFMYACSIVIFLGEVAMGVIEKRRRAKRFNCKCPW

>SexiIR8

MKHVLIWFSIVHSALSDFTFTDFSNCSSASNLIKAAVNISSTYYNSHFTTTVIWKQNSSNCVNGFLMSYPGSVVLSPWATYNDSKVRDINETVGFKQTIYFANDLVEYEIIMKLINEVIRFPIRFILVLENIVTSNQELYDFIEVTSKNDQADLIVVSEMNTGEVKLSTFFPYSEGLCGNYTPVFMKHGQNMFPKKFSNFYQCPIRTALLEYFPYVTVQAEEGRITSVGGYDGKILMIILHKLNASLDVTSAYNNSIFGTYVNGTATGSIGDLATEKADILIPADILTEKRYTVTLPSHTYHTVDIRWVGRRQREVYDWLKFIIPDQTNFTYLHGLVYISFLISASLVIKCKPHLTSATNRVLYQSFIILLGQSVKFVTRSFLLNTLFVLWIWFCFFFRIDYQADLVNALQTPDLEPPFESVEDAVKKVDGYGGVEVVVEYYKDTPLEHNYKVIPMNELRTYIRRIFDGENFLLATDIALVNLLAPYVQILEKRISATGASFYMRPGWPAAKDVDDVIFSLVEAGVIDKILSDGNNHKWILDKKDINDEVIPIGIAFSKINTLFYGLIFMWVVCGIILLIEIKHFKKNIKKVQRK

>SexiIR9

MLVPPMSFALELLLNTILTQYLESSYCVTIFSDKPLSFLFSKSFIYLIPDEENLVEQIYNVSETGCSDYIIRMRDPQNFMTAFERVVHIGNVRRSDRKVIILPYNEEYNEYSDENLPSLIFSMKGSEYLANMLMVVNHKSNGSDCKEFDLITHQFVGPDVESNLPKYLDRWNSCSQQFENDANLFPHDMTNLFGKTLRVACFTYKPYALLDIDTAIEPLGRDGVEIRIVDEFCRWVNCTVEVVREDVDQWGEIYKNESGGIGVIGSVVEDRADLGITALYSWYEEYRVMDFSVAGVRTAITCIAPAPRLLSSWEMPLMPFTWYMWLAVVFTYFYASTGILTAQGCNSSSYPFLNTFGMMIGQSQYEGKPSWKIRSVTGWLLIAGLILSSAYGAGLASTFTVPRYEPSIDTVQDIVDRKMEWGATHDAWIFSLTLSTEPLVKELVSQFRIYSFDELKRKSFTRSMAYSIEKLPAGNFAIGEYVTQEAILDMMVMLEDFYYEQCVVMMRKSSPYTEKVSQLIGRLHQSGLLLAWETQVALKHLNYKVQVEVRLSRSKNDVGTTKALNLGNVMGIFIVYAIGLMVSIATFLGELYAYHRKQKQERIHVD

>SexiIR10

EVPWFCVVFFGYYVVFGADVIVEYYPSQSVLDMNHKVVRKREVNITDDRNVSTNDTGSGIHWRHFNNEPDDGDIHKRALDPIFHGHPKTREELWNERFLNETSSFDQTPSLVNLLHNITLTYLKDCTPVILYDNQVMSKESYLVQNLLKGFPTTFIHGFINDNGELIEPELINAKIDCQHYILFLSDIKISAKILGKQPDNKIIIIARSSQWAVQEFLASATSRNFVNLLVVGQSFKEGDDTKLESPYILYTHKLYTDGLGASQPVVLNSWTHGKFSREVNLFPVKMTEGYAGHRFVVAAANQPPFVFRRIKADLDGGNPRVVWDGIELRLIKLLAERNNFSIEIIEPREPNLGPGDAVAKEITTGRADIGIAGMYLTNDRIRDMDMSLAHSQDCAVFITLMSTALPRYRAILGPFHWHVWVALTFTYLFGMFPLAFSDKHTLRHLLHNSGEIENMFWYVFGTFTNCFTFLGKNSWSKTNKITTRLLIGWYWIFTIIITSCYTGSIIAFVTLPVFPETVDTIKQLLAGFYRVGTLDRGGWEKWFLNSSDPKTNKLLRKLELVPNVEAGIRNTTKAFFWPYAFLGSKAELEYIVQANYTASKSKRAVLHISNKCFVPFGITIGFPNNSVYSAKMNLDISKMIQGGLIDKITNEVRFEMQRSPTGTLLAAGSGTIKIPSAEEKGLTLEDTQGMFLLLAAGFTIAATALVSEWMGGFTRRCRFKKKSDTPTSADSREKFIITPKTDVDSEIKLIEDTESRLHFGSRPSTTVSVDTLEGQVIHVTESSIDVHNTFNVDRFDSRRSSSLDLDREVREIFEKDQKRRRIFSRDMESLDENGSTVSRAAFGDSVKNDI

>SexiIR11

MEKAIWFFVALLFSSVNGDPDQANMIVDIIQSANRPSSVIGKLCWTPTKIIQLYSALVQKNIQFSANPGLKNDYMQFHDEEQHIVILADLNCPDIEEYFEMNSTRIFFRAPFRWILFGDSRAVNEDIVPEAIANVDVLIDSEVLVLRSIDDVYEMHFIYRISSNNTWNIEYYGSWDTKNRFQKSNRFLETTSLRRLDINEYEISICYVLTNNNSINHLSDGLDDHVDTITKVNFPTTNHLLDFLNAKRKYIFADTWGYKVNGTWNGMTGYLVRGEVEIGGSPMFFTFERVSIVDYISSPTPTRSKFVFQQPKLSYENNLFLLPFNTTVWYCTIALVFIIYLVLLLVTKWEWKKTKHSIETREKDAGVLRANVVDIIILIFGAACQQGSPSELKGSLGRIVMLVLFLALMFLYTSYSANIVALLQSSSSHIKTLDDLLHSRIKFGVHDTVFNRYYFSTATEPVRKAIYEKKVAPPGTTPRFMTMDEGVIQMRKGLFAFHMETGVGYKFVGKYFNEGEKCGLREIQYLQVIDPWLAVRKDTPFREMFKIGTKRIQEHGLQYRENRLMYEKRPKCSGGGSNFVSVSMVDCYPAILILSYGTIVALCFLGLEILVHKREKVLRKLKCIKDENYE

>SexiIR12

MKTVQELVDSGIKFGGFGVLRDLFYNSSDPFDNLIGEKWVSIENITEALIDVAVHRNFSVLCSRLELSHISAVTPQLSDTTGNYNYFTFPDNVFSVPIETIALRGFPFMLKFSTTITLFKQSGINEALRYQFKEFTERRRARQLRDLLKEKSDVSPLSAKHLQGGFFALFLGYVSGIFILIAEVLVKTGFFQKKFALFKRNFNLVK

>SexiIR13

MISTPRGYYNGSVVDVRPYRELYRRRRDMRGHSITMSNVIQDSNTTRVHLPREDRLELQYDSITKACWSAAKIGFEMINATPRYIFSYRYGYKVNGQWSGMIADLYANKADVGTNCVIFRDRFDVVTYTDLVAPMRMLFIFRQPPLAYVANVFYLPFSTRVWVTIAVCTAIATITLFLASKVEMVMTKSTTQQQLDGGICDALLLTMSAVTQQGCYLEPRRAPGRMMVFVLFTALMALYAAYSANIVVLLQAPSDSIRSLPQLANAKITLAANDVDYNHFVFNQSREPLYTSIRDRVFPVNGKAKLYSLADGVEKIRQGLFALHSVAEPVYRQIEATFLESEKCDIATVDYLVTFDSFTPVRKGSPYLELIRVVHKQIRESGIQSAIRRRYLVSKPHCTTKMSSFSSVGLLDMRPVLILMLYGVAVSVIIVIGEIIAHKLINRYYKQKSKVQMVKTIRY

>SexiIR14

MFQRTTDPVAIELYHKKVATGTHYNFFTPEEGIALVKKGGFAFHIDTTFAFPLIKATFTEREICETNLVQMYPLQRMGVVVRKHSPYKEHIAYAIRKMYEVGLPPRIQSEIDEPMPECAHTPDSSIFCVGIREFSTPLLALAIGMITSIIVLFCEIIFDRVVKINSVRDFRH

>SexiIR15

MSTRTVFLLIYFIRITFGQTTQNINVLLINEESNALAEKAFEVAKEYVRRNPSLGLAVDPVIVVGNRTDAKVFLENVCRKYNDMLSAKKTPHVVLDFTMTGVGSETIKSFTEALSLPTISGSFGQVGDLRQWRSLNANQTRFLLQVMPPADILPEAIRAIVTKQDITNAAIIFDEFFVMDHKYKSLLQNIPTRHVITPVKSFEANEIKTQLESLRNLDIVNFFIVGSLRTIKNVLDAADKNQYFGRKTAWFALSLEKGDISCGCKNATIVHIRPTPDANSRDRLGKIKTTYSMNGEPEITSAFYFDLSLRTFLTIKSLLDSGKWPNDMKYITCDDYDGKNTPNRTLDLKTAFQEIKETPTYAPFYIPPDDPMNGRSYMEFSTDLLAITVKDGASISSHSLGSWKAGLSSNLTLTDPNNMSNYSAQLVYRIVTVEQKPFIIRDDQAPKGFKGYCIDLIEEIRAIVKFDYEITLAPDGNFGTMDENGNWNGIIKELVDKKADIGLSSLSVMAERENVVDFTVPYYDLVGITIMMKLPRTPTSLFKFLTVLENDVWLSILAAYFFTSFLMWVFDKWSPYSYQNNREKYKEDEEKREFTLKECLWFCMTSLTPQGGGEAPKNLSGRLLAATWWLFGFIIIASYTANLAAFLTVSRLDTPIESLDDLSKQYKIQYAPLNGSAAMTYFQRMANIEEKFYEIWKEMSLNDSLKEVERAKLAVWDYPVSDKYSKMWQAMEEAVLPNTIEEAIQRVRDSKSSSEGFAWLGDATDVKYHVMTSCDLQSVGDEFSRKPYAIAVQQGSPLKDQFNNAILQLLNKRKLEKLKEIWWNNNPESMKCEKQDDQSDGISIQNIGGVFIVIFMGIGLACVTLGVEYWWYKWRKRPAVGDVTQVEPAKLTRNNVDKQGDGFNFRGRNLGINFKPKF

>SexiIR16

KHKVVSSIIILACWPPSDQIKFTRELSQFGMSSMFSCDPAILDHIRHHYLQGVIYITHEHDDMPMFKKMKGIHFMMKYKWLIVGDHVPDALRKIRYDSDMAFLECNFVRNNGTRDLTALTLETATPVHIYDAYVHPRDGISINLWAHWTNASGLVLTHERERILRRLDLKKYPLRIATPVGHYSSDKYDGTFVDFLEDESISDQDPGIRSGYGTSMLLTEVVNAQDVLIENELWAAMINNNSMYVMLTTGEADLSGAILRILYERTYTLDYVMPIWPFRVGFTYLSERESSSNMYLEPFSPGVWWSCLAMMAILALVERVTSKTPMEKDGAFYTVLTTWLQQDASAVPVGASGRCAFTVLSVSAMLVHAYYTSAIVSALMSTGRGGPDSLRALGDSKYAIASEDYDYMRYLFFDVETTWDDLEYLKKKKKTSKFYQELKRGVELIRQGSTAFHSEYNQIYPHFKTFSDEQICKLQHVDTIPETLTWIISTKDGQWTEVLRTAGGWLLETGLGKRLVARLRIPQPPCRASLLAERVKLGDIASLLALTIFGAFLSVVLLGVEILVAKAKNSNLPEGDSKVDNVDDIPNSVEHSNYIE

>SexiIR17

MIGDIYRGEADLCGMVTFITKERMTILEYLTHPTPITLKFVFRQPPLSYQNNLYLLPFSTGVWICTGAFIVILIAILYINTRWDIKKYEDYNKQKIDQSCLPPTWSDITIFVLSAISQQGSSNELKGTLGRLVMFLVFLAFVFLYTSYSANIVVLLQSTSNQIRTLSDLLHSRLELGLERASFNKFYFSSAYTADDPIKKALVETKIAPKGVLTNVMDIEQGVRTMQKKPFAFNMNTGTGYRIVSAIFQEHEKCGLQEIEYITNSNPWLCSRRLSPYGELFKVGYIRIQEHGLSDRENRLIYAKKPACTVMGGSFDSVNMVDFYPVCLILLYGMILAFLLLGIEIFVHRKQMKIRNQLQVE

>SexiIR18

NTGKFIIVCESPVSNECDEQDIVDMCWNYRIVNIVFIRLEGTEPIGFTYYPVADGICNNLKPIKLDYHNNYTHTSYGEVFNNKFRNLNFCPLTVSTFIQPPFIMNITNGTPIGSDGDLLQLLIYGLNATLKMMTPSRGTGWGWREKNGTWMGSLADVKDELANFSMTSAALTLARFTDFQISYSYLVTKVVWVTHPAQIQNVALKLLHPFEEDARIA

>SexiIR19

IFTQERMDVVDYIAMVGTTAVRFVFREPPLSYISNIFTLPFSGTVWLAIFICVLGCSIFLYIASRWEASMGMHPLQLDGSWADVLILIIGAVLQQGCTLEPRYGAGRCVTLILFIALTVLYAAYSANIVVLLRAPSSSVRSLPDLLNSPLKLGASDFEYNRYFFKKLNDPIRKAIYEKKIAPKGKQANFYSMKDGVERIRKGLFAFHMELNPGYRLIQETYQEDEKCDLVEIDYINEIDPWVPGQKRSPFKDLFKINFLKIRESGVQANVHQRLTVARPRCSGHMSTFSSVGITDMYPAMLMTLYGMLLAPAVLLLEITYKRLITIRQQKRILADSDHIPFRH

>SexiIR20

NTYDHCLSVDFCSLCLQESPEKSTQQLYRHKILPQGERAYLSVVDGIARVRTGFFAFQVEKSSGYDIIKQTFTEREKCSLSEIEAFKPPLVAVPMKKHSGYRELFATRMRWQREVGMMDRARRVWLVARPRCEAAGTGFVSIGLIDVLPALQVLLRSAHHSIIVVCSASPLLLLR

>SexiIR21

EIFFSLWYHKAINTIMVQYNDVEDTFLVSNYTPYVNEKYEVQPSNKFGCWTRRNLGIPVVGFERGLTCVEGCHNVTILSRLRAQHLGTCIGFDTIPIKSIQDLKGLILFEDRSKNLHGFMFRSYVIEVKPFSIIDVHEDGSYTFRARDGMIWSTMSKLMNFGIDLSPSVSFMKKPFNFEVNIEKIFSFARRKGDLCLFPIYQFDVIVVEIDFTFPFKESGICIAAGRAGFETTLFEMKNLTVNLKYLFVFHGCFVCIWMVFSLYKAAEKGRFIWDQIGKDFMNAWRNVLMLNLCNPPKYESFRIFLTICLWCFFVLNFTTQAKIISFFTAAKRGKEVDTFEDVIEKGYPIEAMASPDLILPDTEEKFRIINSRLVYEQDIYGCITRMVNDNRRFCLIDCSVGRYIKRNKLNNKGEQYLHIAERDRIHSHYLTMVLSKHSPLTDRYNRYMMMIFEAGLVRKWEQYRYTDIKDEVTTKALSIQDLSGVFKVYCFYLGLTLVSFVFELAIPAIESCQKKITILRKR

>SexiIR22

VWPLVFATLLVTGPALWIVIAAQSLWQKRKCDQLSLFSTCCWFTTTLFLRQSSSKEPSSTHKARLVSVLISLGATYVIGDMYSANLTSLIAKPSRERPIGTLAALEEAMRDYGYELVVESHSSSLAILENGTGVYGRLANLMRRQRVQRVRNVEVGVRLVLSHKRVAVLGGRETLYYDTERFGSHNFHLSEKLYTRYSAIALQIGCPYLETVNNVVMTLFEAGIIAKMTTDEYKNLPEHARRSDPVTESDKQGGEVMGESATASSQTPQGESTKGLQPVSLRMLRGAFCLLGIGHLLAAISLAVEIQLHRRSKRRRKPAQNEHRKAQKLLMLGKSVMLFKRGCKKVCTSVFTSIDKALGSDVKD

**GR**

>SexiGR1

MIFIYLMKLDFLFMYLITVDMVLHLTNVARIIMFEILWRQMADLRRNFEHDLSIARRFEHGEEIMMEKIKKCLNDYTKLLDVLNEKSGVTKFWIFLSLSIAIPRLVDIVFFSLSSGPEVEVTLAYKLILTETLMNTVVLLAPAALAEMITNEIDVLRLFVIKQLLVCKGETNRDEILNALVYLEQHPFKYTVWRLFTVDGNLILSVVNLFTTYILAMVQFAHIFD-MLLQLTKVCFSFVLLYNLIKDFNPILSVGEPCIGSNR

>SexiGR2

MSIFTKYTRVIIVSNFIIRLVITVIHIYIRRVSSLIFLIVTFALMSVDINRIFNLLIFSIIQNRIKQLKLFLKSNSVYVNVTGRNEIETSIKNVRMSLLYYNNILDNLRSRTSKPLQVLVNYLSFLIPISLVRMLVPFFS

>SexiGR3

MVPSVASITFDINIVYASFILSLLRRLVISWIEEFQSNGDSNNECYWNAMFDLYVNILEAYKLLKITSSLQVVYYTIHTVCHGLMTEVILLQMRHIIPETQVKFTMFAMLSQMWLVKNVALQIVLCVKSERFYAAMEQVPSVCVELMQTRGCTDKQRQVCKNIQRLHETSFKKMSGFGLFVVDVALPLRIASVLTSYTLAILQFALS

>SexiGR4

MLVRRMDVALGGIIMLSCSCNFYFICLQMFLGITQGLSTDLMTGIYYVVSLAWLCIRVISVVLSASGVHTHSKTALKYLYTYETNCYNIEVGRLQDQLTKDYIALSGMGFFYLNKTILLQMAGAIITYELILIQFDGQGQIDSNSTLNIPLN

>SexiGR5

MCIRFDFNAIYATRCLGLMTDSLKILTANLQMSTYISDSLDKNFWSAMFGCYVDITEVFQIVEKTFEPMILFYLVHTCSNSLLDIWVRVKLRPTVPNNELKYTLLTCSSLTWVMKNLLIITFLCVECEKYYSAIKEIKWMCTQMTASERSSANQKTFCRNILRVQDATFKKLRICGLFAVDASLPLRVIAFITTYTIVL

>SexiGR6

MLNCRKRRNRNSYYHVEVTTDNLVDEDVQSMLRPLNLMTNIFCTPLYRIKNGFITPNSYITNVLSFWFLAMSLSLFFYRAYDRIRSRIDVVKLTSVIHFDFLTLVIVALATIFRHSIIVFYSKNNVLFVLNFVKLNRFLHDEIDCKRLIFG

>SexiGR7

MTLNLTPLKYLLFIENVTCVFRNYSHFKGWSRILAFAWFLLEVLSIFINALVNFALQNEIHIKAQRIYFFTSIGFSLYVMVAAVYYSKKFYSLLLNFDGFYDIFDDRIYEHKLLKAQKVLTISIVLFCLVKIVTSSAVRLSNQKSAEIMYESILYNYNFNLSDFRYLFQYFVLYSILFVICEQLRTITRSIDRELSPPRDMRENVEQAGDLSSETVSHDKINKWVKAYENINDTSNLCNAMFSIQLTVMILIVTVYYIILMYSIALISVEGSQTMPTSMFAHLFSMAIFLTALFVISRAGQNVQNSSLQLRQKLCELCVYTLDNEECYKLAKDLLRCVRTRPVRIHVFGTLDVDMSMLPSIVAFFTTYTVIALQFNNVL

>SexiGR8

LIFSYSRALSSPFYLLYYLYSFVFIVLRALMLSLFASNVHCAALEPVYSVYDVPSTVYDNEVRRFQLQLHYTEVGLSGKFFYVTRNMILKVMGTIITYEIVLLQYSVTPNPYYNVSSTLAIIVDNVTHSYG

>SexiGR9

MSVIFYLIFTVYLFCMIFVTENKGGMEIFEYFVGNVYHFILFSIGGFINFFTNIKQKYNNVLYVLKIQNVHRILKLNETCFKRTIISNWICVIAINCFHIYGALMYSIAYREVNYFNSLASYSNIRFDVNVIYAIKCLKLMTDSLKVWTSILQKSTYSDDSPNNCHWKAMFECYVDIVEIFIIVEKTFHPMVFFYLIHTCSNALMEIWSRVERRPIASNNELQYTLLSFT

>SexiGR10

MTVAFKSELAIMTIPDHLFDEGINNTLFHHDMRHIQQNKIVYEKTQRDYEQEQRDLLSSQDGDTCEIHDQFYRDHKLLLVLFRALAVMPITRSRPGTITFSWKSRATMYAVCFYIAATAVVLIVGYERIMILRSIRRFDDYIYAILFVIFLVPHFWIPFVGWGVAHQVAIYKTNWGKFQVRYYRVTGENLQFPNLKTTIVIISVGCLLLAVCFLLSLCILMDGFLLWHTSAYYHIITMINMNCALWYINCKGIKIASQSLSACFRRDVEQECSAKLISRYRYLWLNLSELLQSLGNAYARTYSTYCLFMFANITIAVYGALSEIVDHGFGFSFKEMGLFVDAAYCSTLLFIFVDCSHKSTLTVAAGVQDTLLSIDVLAVDRPTQKEIDHFIQAIEMNPAVVSLKGYAHVNRELLTSAISMIAIYLIVLLQFKISLPKDPQIVAT

>SexiGR11

MSFDINMLYFTFIMKLLSMTLCVWTENVRKSINSYKDCYWKSMFDVYMNIFEVYEIVVKTFGYLVLFFTLFAVSHGLMNIGVVIKMKLAMAGAGIRVSIFIFIIQVWFLKNIALIAYLSIVCERFYEAIEDVHGSFVMLTKRHGRSDTQRRVCKNIERIQRARFKKLNACGVFTVDARLPLDLIRFTTTYTIVLLQFSI

>SexiGR12

MPRVFFVQNKVDIAKQKSPRSITPFFDALRCTLIVGQVFSLLPFVGVFSNVASNVKFIKTSWKCVYSLISLIGQIFMAVLCINKLARSNVTLNGTSPVIFYVTTCVTMIMFFHVARRWPQLVQHIAKAEDMDPNFDCSLARKCNITCAVVLLLALLEHILSLLSAFAGAAACYSGMDTYEGFVTHFYPWVFSYLPYSPVLGIITQFLHFQSTFIWNFSDLFVICMSYYLTSRLEQVNKKLLAAQGKYLPEIFWRSTREDYCRATQIVRKVDDVISGVVFISFANNLFFICLQLFNTLEDGLKGTGECSNKGKKIVVSKNGPLGGHEAATYFLFSLVYLLSRSVAVSLIASQVNSASAVPATVLYDVPSPVYCVEVQRFLDQVNGDKVALSGLQFFSVTKGLLLTVAGTIVTYELVMFQFNSSTPTLNITSPTVLTPSSPSLTTLSS

>SexiGR13

MNKENGFRVYNPNPVNKETRKREMFQRIDEKDGIKEYDAKDLYGPEITDKDGALLDAHDSFYITTKSLLVLFQIMGVMPIMRVPKHAQTTKRTTFNWISKATLWAYLVWGLECIIVVKVGRERLANFQNSSNKRFDEVIYNIIFLSILIPHFLLPIASWRHGPQVAIFKNMWTHYQLKYLKITGTPIVFPNLYSLTWGLCVFSWGLSFAVILSQHYLQDDFELWHSFAYYHIIAMLDGFCSLWYINCNAFGTASRGLAMNLHKALGAEHPALKVAQYRHLWVDLSHMMQQLGRAYSNMYGIYCMVIFFTTTISLYGALSEILEHGLSYKEMGLFVIVGYCMTLLYIICNEAYHASRKVGLEFQVRLLNVNLGAIDRSTQREVEMFLVAISKNPPIMNLDGFTNINRELFTANVSFMSTYLIVLMQFKLTLLRQSARKTLKSIVKAVFNTSTTMLDDDFDEEDEE

>SexiGR14

MHARAVDRSSRDTFYTLAPLNCVLRLFNLSCIHRKNNQLMISWSMMKSVIYVFVFGFFNFFFLLNKIQRLDVIRSNTFLVTFSDMIQMTYLIGYFAYTVDLFYVYKYGRDGFLKYFKTFDHIDQILGMTYYDAIRKIIVRILGMCILFFITSSAVDYMCWVDGFGWIAPTLYSLDYFYFFLSTLMAIDGASHIVQMEYRLKLIKELLQGYYNPLSTKTVPNPNKVWAVRNDMGNVQLESLKTLSHGRFIEAIGFNRCYLLLLDQTHYINSKFGVRILIFCVNLLANMINLLNVSIRFATGSMVSPKGISHLPTFSSLLRFMNWSMVGISMVLHCEKVYREEEKIVEVIDLILVNKVNSSTLTETLMNFRNLLLTRPIKFHASHFFTVRYPLLVSVISSAVTYTIIMLQSIK

>SexiGR15

MFNIDSISNNNRSSYNSKFLKLLLPLRCMQHAIFLSNFTIQHNRIKAHSMRYYIATFICILGLIIYRFLIDTQNLIYKHFSLVIRIINKNVPHLWLVVNITFFFLNFIKRQDHVVMLWMIQRSFKELNYKHYSRTTICNWIVVSCHVIFLFFIIFAFLKINKIFIAISFMSLDIQITITMQLIHLIREGVLTWISEVNYYSKHVEYEEEKYNERMKIMFQVYLDLMKAFELFKGIHQFLNFFFIADLFLFSLSFVQETIEICKYHIPDDQQFHIMVWLAAVCFWLTRRTVFIVLLCTLCEKYYMTISDADAYCSCLLNRFQETVAMKRLCKNVLRLNRAAFHKMRAYHVFTIDGRLPQKFCCFLFGHIIVLLQFAFL

>SexiGR16

MGLSSRYKRLNLYVRNVVTVEKRSEAKPKFGTDLYLQIQVWRRLREAYVRQSTLVRMVDRKLGSLVLLSNINNLYFICLQIYLGIHKPSSSTISRCYFLFSLSWLILRACSVVIAASDVHLHSQRALKLLHSCPSANYNIEIKRLQYQLAHDFVALTGMGFFSLRRELLLEVAAAILKYELVLIQYDK

>SexiGR17

LNEKKYDDFLPILNNVFKKARYFGISGYGCSLAFGWSLILFAMLVVAETVAVWKVVRFLRGWLMSASDNGLIGRMAGAIFYANALISLFFSSKFVHSWRNLYTYWLSMETNTALKFPPDVRMKKMAIFIIVFVISVASVEHILSMISATGVGFPIEEFVYRYVTLSHGFLLRALSRVRAQ

>SexiGR18

MKAQQNDNEKHEWILLRQMNEHDKLMNIVIRISNNMLMFYNVSRLLLQVHIFLLTVTCKMSSYERTSSNRINALVDHTRRKPFHIFSKDENNILCQHIVEPYLESILRPLNIIQNIFFCAKYRIRGHVIKPISRLYSAVSGIFYLLFNAYLYYMFFETQNEDNFEHFVMNVYDYVLFSIGGFVNFFINIQQKYNNVLYVLKIQNVHKILKLDETCFKRSIIYSWVCVIAINCFHVCWTLIYYFCFNDIDILNTLAAYANIRFDVNLIYATWCLRLMTESLKFWTYGLQNSAYTNDALDNKHWYNTMFGCYVDITENFQIVEKTFQHVVSFLHVKNLRLK

>SexiGR19

MEKVMNLYHNMIKSYNYLIEAVKWQLLVTIVSAFLNILSCCYRVSLTIIKEDVPLTFMITYVGLLAGIMLPLFSPCMLGDQVHDEVRRLRELLASRLYENQLDKSSRGMARALLAWTETRDLSFSLLRMLDIDISLPFKFVGLLVTYLIILLQFQKVINP

>SexiGR20

MVKVKEMNSKLENTRVYKKALCNVIYSIKPLLFMEYLYGIYRFYFTSGELRLCNRKMKSYSILTILSFLFTVIVSIDFPVFMSGSVKSVDVMEEVPAFVVLIQFTTSTITASFLVNSANIAIFNNLAKIDAVLDAESISDYYKRSRRETYAFIFVLLLLHLINIIIELVTAEQITLHALFVLPLYYIQKLEIAVFCKYMLMVKRRLVLINDHLKVFVQEQEKKNKTIFCVSKSVLNSKEKIEINFIGRAADDNTKIRDLAAMYDVIGRICSMINEVFNFQIFMTLVSTFTYVVITIWLSLYYYRTPASHSGELINTAIWCFSAIYTVGAMSFACERLLLARNETRVQVNKIIMNYDLPNPMRVQAKAFMELVEAWSLRIYIYDLYSVDITLMLKFISVATTYLIVIIQISHFV

>SexiGR21

MDTNTLYSIRLINILRHKIHLWNRKVFELRRVQDGGPQKGQYKKMFQAYFDLLRCYDIYKRFFQLVILCHYVSCFVNNLIYVEMSLKKSPMLHQGMDQLLTVTTNVVWTLKNLILEVVLCIEFEKFYIASQETQDICVNMLKGNCLSDEIQLYKNVRRLHRAKFNKMNVCGLFYVDAKLPRKMIALVANYTIVLLQFAFL

>SexiGR22

MLNISKDLNVVDSGYASLFWLSGCLFYSLCEYIGCYFYLSFFIDRTVYCFYIMMLSHDCEQILFFVLLRTIYARLSIIKAHVLKVFSAETRTDSYRRKLNKVEALSNNAQLDISSLHRVYDLLHKCAEQLNSIMSLSMIVILLTAGLSTTMLLKMMVKVIQTVTVSNTFGKSTIIYLFGRGMKYTGLVVVPCYYSRVTSAQVATIRTCLHDAVNSKPLDKIERRKVKAFFQLTRENEFTYALGGVIRLNMSLPLSYMSLCTTYLVISIQFSKFFD

>SexiGR23

LFILFTYNLLNLLLDIFTETNYTWAIVVFHIYTFQKLVVLSFFNHIEMITCRLEVINNCLKTFVFEQDIKQTTIFFTKSRKKEMEYKINFIGRPTENNMKIRELARMYNITGKITFLINDVFNVHLFSILVAAFAYIIVTIWTALYIYRSLEFSVKDIVTLAIWCIGAFINPAIFAFVCEYLLRARNETKVFVNKIIMNYDLPRTMRVQAKVFMELIEAWPLRIYVYDMFSVDITMILK

>SexiGR24

TANFEFIVCKINFKKMYKSLIVLCVVYCVVIEARPKWQVLPPMPGYVPVYIRPGDTPLEEINLDLAEAFHELPSGRSVGKQVEASPEAPEQADQPQPDEVPAAPADEIVDHRPYEKKILEKKKKTDSEIPKPIERR

>SexiGR25

MSFYTSNSLFPTTVNIPNGFPVQIDEKPKNKIIFLDATPVRTPVKPISPNAVAPMRNNLVEPHISNDIIYENIKPVFTVLRIMGVLPITRPASCINQFQIASASMLYSVLVFCSLVSYVLYLSLHKVQILRTAEGKFEEAVIEYLFTVYLFPMIAVPILWYETRKIADVLNGWVDFEVVYKQLSGRTLPVKLYKKSLAIAIIIPILSTTTVIVTHITMVHFKPMQLVPYVFLEILTYMLGGYWYLLCETLSICANILAEDFQAALRHIGPAGRVAEYRALWLRLSKLSRDTGIANCYTFTFVNLYLFLIITLSIYGLLSQISEGFGIKDIGLALTAIYGIFLLFFICDEAHYASHNVRTNFQKKLLMVELSWMNTDAQTEVNMFLRATEMNPSQISLGGFFNVNRTLFKSLLATMVTYLVVLLQFQISIPDDSQSQDDEEDAPLNITSATTEALTTTTTVMTTILTTLAKKKKKN

>SexiGR26

MKAYGFFIFFLYSALFFWLLFFVPDTEAANSSEQSDLITTVDSIPFFVVLFQYAALIVSTNFFSSMNIRMITLLAEVDKTLQLEICTDFYKKMSSRLSKFLIFLTISHVINGLVDFITVTDRAWALTVFPLYFLQRFEVFVFCAYVIIVNGRLAVINNYLKEFNQEQDKKMVTVFTVKDTKIKTEKYFNYIGRPSIRNMKIRDLATTYDNIGEFCFMMNDVFNFQIFLTLVSAFVYIVITIWTSLSYYRATTYSANTLINNAIWCFNTICNVATMSFTCERLLISRNETRILVNKIIMNYDLPKTMRVQAKAFMELVEAWPLRIYVYDMFSIDIKLMLKFISVATTYLIVIIQISHFV

>SexiGR27

MRNLWNTVISNIGVGSVNTVNSLFKSWGKIAPTYTMDLYSLEKFKKYKKDWSYPVNIKHQEQVIPAKEKPCSTFQSAMKVTLTIGQCFGLNPVQGIRGDDPAKLRFKLLSWRCAYTGISLVGQFTMAFVLFLSLFKEASSTVDTATALLFYSFGFTTTILFFRIATKWPKLCMVIAKAESADPNTDAKLKRNFNIACGIILSLAFLEHGFSELHGISIALDCHPEKPLYETFMRDSFAWLFLYLPYNDFIGFLSHFFNIQCTFNWNFSDVFVICMSMYLTARLEQVNQRIIAAKDKNSPSSFWRTMREDYNRSVHLVRQVDKIIGGVVFISFASNLFFVCSQLLHTLAGGIRAAPKCRPDLNTDRRFFGGYEHAVYFAFSFLFLVVRSLTVSLTASKVHAASIEPAHALYDVSSANYCVEVERFLDQIHGDTVALSGLQFFHVKRGLILTIAGTIVTYELVLMQFTGITPSSPEPG

>SexiGR28

MVLVVISQLATSILDFVAIGDVAWAIAATPLNFIQKLEIITFCKYVDFLRRRLLMINSYLKTFVDEQDQETTTVFTIRSRTMETTEKFNFIGRASDSNTKIRDTAKMYDVIGQICTMVNEVFNFQILTILMSTFAFIIIIMWTSLYYYRSAISDLSLLMNVIVSSFFWICYVAIMSIACERLLLVRNETKILVNKVIMNYDLPKTMRVQAKAFMELVEAWTLRIYIYDMFSVDITLMLKFISVATTYLIVVIQIFNFF

>SexiGR29

MSVVLNIGLKMIRPNWISQNNYSLSLSPIARVIYGVESAKVEEVTAAPVPTESEARSSRPTHCVVGGAHAFILRISSFFGLAPLRFEARNNGFTVSISSAMCVYSYILVTVLVICTIFGLVAEINVGVELSVRMSSRMSQVVSTCDVLVVVATAGAGVYGAPQRMRNMLKFMENIASVDTSIGGQYSLLTERKLCGIILAILIFFSVLIADDFTFYALQAKKLDREWDVVTNYLGFYLLWFVVLMLELQFAFTALSVRARFSAVNDALALTARQVSVPVEKPKSPSSLNIYAIRVAPADSARSANVSLLVETMTGREHVVIIKRTVSIISASGEPRLIVSPCDAVRRLAALHGTLCDVVNSIDDSYGLPLIVVLISTLLHLIVTPYFLIMEIIVSTNRVHFLVLQFLWCVTHMLRMIVVVEPGHYTIAEGKRTEALVCRLMTSAPSTGVLPSRLEIFSRQLMLQSVSYAPMGMCTLHRPLVASVIGAVTTYLVILIQFQRYDS

>SexiGR30

MSKANYGQFLRNTNLILPEQPNHDEFLIAMENVFRLSCIIGVLGSKKYICYVWSAFILTVLLVMESQAIWKVIKALAGIAIDTAGQRSVTARLAGTIFYTIAILSLILASKLFRSWGELSALWVRVERIMAVKVPSDGTLKRRMYIIIGFMTVCSLLEHLLSIVSAIGLDCPSHLIMKRYILISHGFMILRHEYSDWYAMPLAFMSTIATMLWNFQDQLIVLISMGLTSRYSRLNQCLANICALEKKQMESDQKTEATKVYTWRKLREAYVKQAMLVRKVDDAIGSIIILSCFCNFYFICLQLFLGITQTQSDEPLRTVYYFTSLGWLCFRVIIVVLAASN
